# Unlocking opioid neuropeptide dynamics with genetically encoded biosensors

**DOI:** 10.1038/s41593-024-01697-1

**Published:** 2024-07-15

**Authors:** Chunyang Dong, Raajaram Gowrishankar, Yihan Jin, Xinyi Jenny He, Achla Gupta, Huikun Wang, Nilüfer Sayar-Atasoy, Rodolfo J. Flores, Karan Mahe, Nikki Tjahjono, Ruqiang Liang, Aaron Marley, Grace Or Mizuno, Darren K. Lo, Qingtao Sun, Jennifer L. Whistler, Bo Li, Ivone Gomes, Mark Von Zastrow, Hugo A. Tejeda, Deniz Atasoy, Lakshmi A. Devi, Michael R. Bruchas, Matthew R. Banghart, Lin Tian

**Affiliations:** 1 Department of Biochemistry and Molecular Medicine, School of Medicine, University of California Davis, Davis, CA USA; 2 Center for the Neurobiology of Addiction, Pain, and Emotion, Departments of Anesthesiology and Pharmacology, University of Washington, Seattle, WA USA; 3 Department of Neurobiology, School of Biological Sciences, University of California, San Diego, La Jolla, CA USA; 4 Department of Pharmacological Sciences, Icahn School of Medicine at Mount Sinai, New York City, NY USA; 5 Unit on Neuromodulation and Synaptic Integration, National Institute of Mental Health, National Institutes of Health, Bethesda, MD USA; 6 Department of Neuroscience and Pharmacology, Iowa Neuroscience Institute, Roy J. and Lucille A. Carver College of Medicine, University of Iowa, Iowa City, IA USA; 7 Department of Pharmacology, University of California San Francisco, San Francisco, CA USA; 8 College of Biological Sciences, University of California Davis, Davis, CA USA; 9 Cold Spring Harbor Laboratory, New York, NY USA; 10 Center for Neuroscience, University of California Davis, Davis, CA USA; 11 Present Address: Max Planck Florida Institute for Neuroscience, Jupiter, FL USA

**Keywords:** Neurotransmitters, Fluorescence imaging

## Abstract

Neuropeptides are ubiquitous in the nervous system. Research into neuropeptides has been limited by a lack of experimental tools that allow for the precise dissection of their complex and diverse dynamics in a circuit-specific manner. Opioid peptides modulate pain, reward and aversion and as such have high clinical relevance. To illuminate the spatiotemporal dynamics of endogenous opioid signaling in the brain, we developed a class of genetically encoded fluorescence sensors based on kappa, delta and mu opioid receptors: κLight, δLight and µLight, respectively. We characterized the pharmacological profiles of these sensors in mammalian cells and in dissociated neurons. We used κLight to identify electrical stimulation parameters that trigger endogenous opioid release and the spatiotemporal scale of dynorphin volume transmission in brain slices. Using in vivo fiber photometry in mice, we demonstrated the utility of these sensors in detecting optogenetically driven opioid release and observed differential opioid release dynamics in response to fearful and rewarding conditions.

## Abstract (web summary)

Dong et al. developed and validated κLight, δLight and µLight, a suite of genetically encoded opioid peptide sensors for probing opioid drugs and brain-region/circuit-specific opioid release in behaving animals.

## Main

Neuropeptides (NPs) are small proteins that modify neural activity, regulate brain states and control blood flow in the nervous system^1–5^. Neurons synthesize and release NPs in addition to fast-acting neurotransmitters (NTs) such as glutamate and GABA^6^. NPs activate select G-protein-coupled receptors to modulate synaptic strength, neuronal excitability and circuit dynamics. Unlike small-molecule NTs, NPs are hypothesized to be released into the extrasynaptic space and thought to be cleared by proteolysis and diffusion over a range of 100 micrometers to millimeters to affect neurons, leading to long-lasting modulatory effects^6–8^. A comprehensive understanding of the conditions that trigger NP release from neurons and the spatiotemporal extent of peptide release has been lacking, and yet is critical for understanding the actions of NPs at the molecular, cellular, circuit and network levels to their influence on animal behavioral states.

Among all known NPs, the opioid system is the most functionally diverse and clinically relevant family^9–15^. The opioid receptor family contains distinct receptor subtypes—kappa, delta and mu (κOR, δOR and µOR, respectively), as well as nociception receptors—which can be activated by at least 20 endogenous opioid peptides with differential affinity and selectivity^12,16,17^. κOR, δOR and µOR and nociception opioid receptors activate inhibitory G_i/o_ G-proteins, which leads to reductions in cellular excitability and NT secretion in receptor-expressing neurons. Opioid peptides and their receptors are widely distributed across cortical and subcortical brain regions^18,19^. It is thought that the diversity of opioid peptides is essential for modulating complex behavior and physiological processes, such as pain, reward, substance abuse/dependence and stress^20^. Opioid drugs targeting these receptors are used to treat severe pain, but prolonged use can lead to addiction and overdose^21^. Newer efforts have isolated opioid receptors as potential targets for anxiety, depression and addiction^22,23^. Some of these efforts have been hindered by a lack of high-resolution methods for studying endogenous NP release in vivo.

Studies into NP systems, especially opioid systems, have been historically challenging due to a lack of sensitive experimental tools in the spatial and temporal domains, which can facilitate understanding the complexity and diversity of NP signaling in a circuit-specific manner. The endogenous opioid peptides have similar structures and bind to different opioid receptors with relatively lower selectivity than some NP molecules at their cognate receptors^16^. Physiologically relevant NP release by neurons is thought to be difficult to trigger, and the released concentration may also be at orders of magnitude lower than classical NTs (nanomolar versus micromolar or even submillimolar)^24^, making it extremely difficult to adequately probe the conditions to trigger the endogenous peptide release and measure the released concentration ex vivo and in vivo^25^. As a result, it has been exceedingly difficult to study the processes that regulate opioid NP release. Recent technological advances have begun to reveal the anatomical and spatiotemporal features of opioid signaling^26,27^. Transcriptomics studies have documented the distribution of opioid peptide–receptor pairs across cell types in the cortex, highlighting the substantial function of opioid signaling in mediating transcellular communication in neural circuits^28^. Features of peptide diffusion and clearance have been revealed by combining light-triggered photorelease of caged enkephalin with electrophysiological measurements of peptide-evoked currents in brain slices^29^. In vivo, optogenetically driven peptide release has been detected using high-speed microdialysis^30^. Despite these successes, it remains challenging to quantify behaviorally relevant endogenous opioid peptide release with subsecond and subregional resolution.

To bridge this gap in technology, we developed a class of genetically encoded opioid peptide indicators, κLight, δLight and µLight, based on κOR, δOR and µOR respectively. We used these sensors to systematically evaluate ligand binding-induced conformational changes at all three receptors and thereby established the binding specificity and efficacy of 14 opioid peptides and 8 opioid drugs. In acute hippocampal slices, we used κLight to determine electrical parameters that can trigger endogenous opioid peptide release and quantified the diffusion rate of dynorphin using photoactivatable peptides. Using optogenetics to stimulate opioid peptide release, we detected circuit-specific endogenous opioid signaling in vivo. Finally, we used these sensors to reveal rapid opioid peptide release in a subregion-specific manner in response to fear and reward conditions within the nucleus accumbens (NAc) of awake, behaving mice.

## Results

### Design and engineering of opioid biosensors

**Fig. 1:**
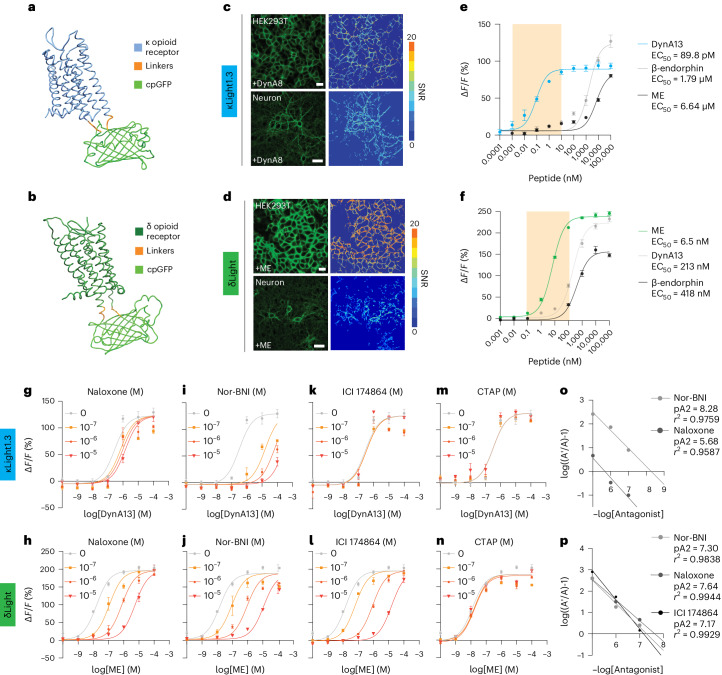
Development of the opioid sensors. **a**,**b**, Simulated structure of κLight (**a**) and δLight (**b**). **c**,**d**, Representative images of four independent transient transfections of κLight1.3 (**c**) and δLight (**d**) in HEK293T cells and cultured hippocampal neurons. Heat map indicates SNR upon addition of DynA8 (100 μM) or ME (100 μM). Scale bar, 20 μm (cells) and 50 μm (neurons). **e**,**f**, In situ titration of κLight1.3 (**e**) and δLight (**f**)-expressing HEK293T cells respond to ligands in a concentration-dependent manner (DynA13, blue; β-endorphin, gray; ME, black). Error bars represent the s.e.m. The highlighted area corresponds to a concentration range from 1 pM to 10 nM or 100 pM to 100 nM. Dyn, dynorphin. **g**,**h**, Schild plot of κLight1.3 (**g**) and δLight (**h**) dose response with 100 nM, 1 μM and 10 μM of naloxone. **i**,**j**, Schild plot of κLight1.3 (**i**) and δLight (**j**) dose response with 100 nM, 1 μM and 10 μM of Nor-BNI. **k**,**l**, Schild plot of κLight1.3 (**k**) and δLight (**l**) dose response with 100 nM, 1 μM and 10 μM of ICI 174864. **m**,**n**, Schild plot of κLight1.3 (**m**) and δLight (**n**) dose response with 100 nM, 1 μM and 10 μM of CTAP. **o**, Combined Schild regression with Nor-BNI and naloxone on κLight1.3. **p**, Combined Schild regression with Nor-BNI, naloxone and ICI 174864 on δLight. **e**–**o**, *n* = 4. Error bars represent the s.e.m.

**Extended Data Fig. 1:**
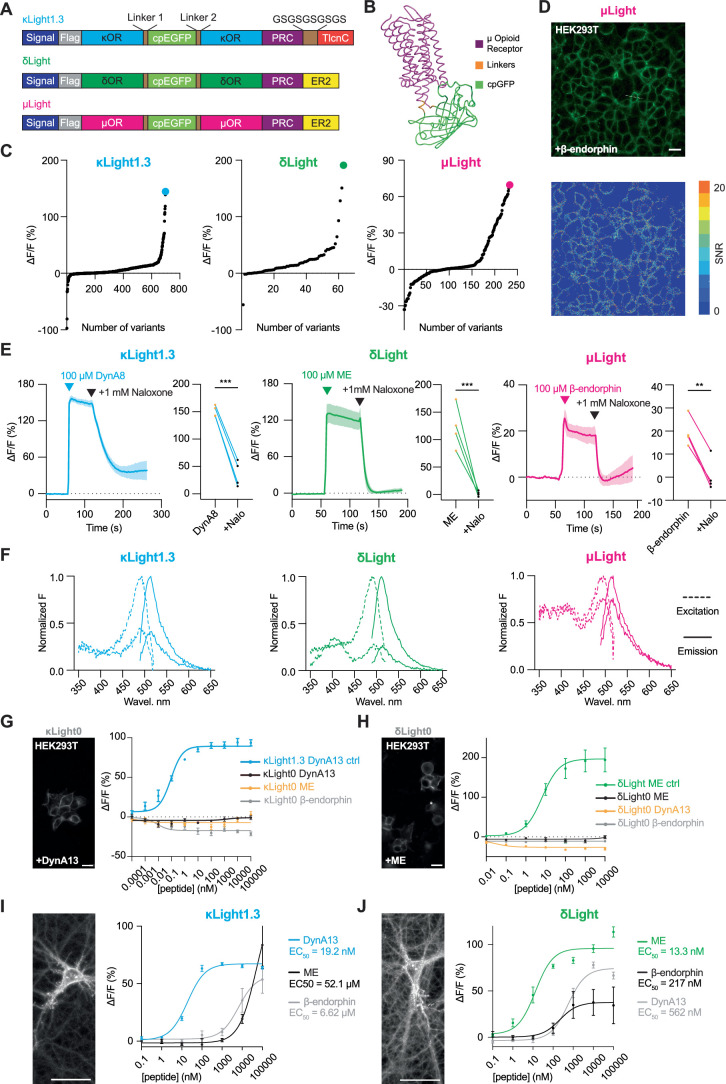
Screening and characterization of the opioid sensors. (**a**) Schematic diagrams showing the components of recombinant DNA for each opioid sensor. (**b**) Simulated structure of µLight. Mu opioid receptor (µOR, magenta), linkers (orange), cpGFP (light green). (**c**) Optimization of the opioid sensor variants. Dots representing ΔF/F (%) of variants screened along optimizations, including cpGFP insertion sites, linker screening, and point mutations. A ligand concentration of 100 μM was used for screening; U50,488 for κLight (blue), ME for δLight (green) and DAMGO for µLight (magenta). Variants with the highest ΔF/F (%) indicated as κLight1.3 (144.47%) with 698 variants screened; δLight (191%) with 63 variants screened; µLight (69.6%) with 233 variants screened. (**d**) (Top) Representative image of 4 independent transient transfection of µLight in HEK293T cells. (Bottom) Heatmap indicating signal-to-noise-ration (SNR) upon addition of 100 μM β-endorphin. Scale bar, 20 μm. (**e**) (Left) Agonist (100 μM DynA8) and antagonist (1 mM naloxone) response of κLight1.3 transiently expressed in dissociated hippocampal neuron cultures. n = 4 wells. Solid blue line represents the mean, and shaded area represents SEM. ΔF/F (%) comparison between two states: agonist response (151 ± 5.0 %), + antagonist response (36.9 ± 11.7 %), two-tailed unpaired t test, ***p = 0.0001. Nalo = Naloxone. (middle) Agonist (100 μM ME) and antagonist (1 mM naloxone) response of δLight transiently expressed in dissociated hippocampal neuron cultures. n = 4 wells. Solid green line represents the mean, and the shaded area represents SEM. ΔF/F (%) comparison between two states: agonist response (123 ± 19.4 %), + antagonist response (1.82 ± 2.4 %), two tailed unpaired t test, ***p = 0.0008. (right) Agonist (100 μM β-endorphin) and antagonist (1 mM naloxone) response of µLight transiently expressed in dissociated hippocampal neuron cultures. n = 4 wells. Solid magenta line represents the mean, and the shaded area represents SEM. ΔF/F (%) comparison between two states: agonist response (19.6 ± 3.2 %), + antagonist response (0.75 ± 3.6 %), two tailed unpaired t test, ***p = 0.0082. (Right). (**f**) Excitation-emission spectra of the three opioid sensors under both bound (upper lines, 100 μM DynA8 for κLight -blue, 100 μM ME for δLight -green, and 100 μM β-endorphin for µLight -magenta) and unbound (lower lines) states. Excitation (dotted lines) of all three sensors peaks at ~490 nm, and emission (solid lines) of all three sensors peaks at ~515 nm. (**g**) (Left) representative imaging showing κLight0-expressing HEK293T cells, scale bar 20 μm. (Right) κLight0-expressing HEK293T cells respond to ligands in a concentration-dependent manner plotted together with κLight1.3 (DynA13-κLight1.3 – blue, DynA13-κLight0 – black, β-endorphin-κLight0 – gray, ME-κLight0 – yellow). Error bars represent SEM. n = 4 wells each. Dyn = dynorphin, ME = met-enkephalin. (**h**) (Left) representative imaging showing δLight0-expressing HEK293T cells, scale bar 20 μm. (Right) δLight0-expressing HEK293T cells respond to ligands in a concentration-dependent manner plotted together with δLight (ME-δLight – green, ME-δLight0 – black, β-endorphin- δLight0 – gray, DynA13- δLight0 – yellow). Error bars represent SEM. n = 4 wells each. Dyn = dynorphin, ME = met-enkephalin. (**I**) (Left) representative imaging showing κLight1.3 - expressing dissociated hippocampal neurons, scale bar 50 μm. (Right) κLight1.3-expressing dissociated hippocampal neurons respond to ligands in a concentration-dependent manner (DynA13 – blue, β-endorphin – gray, ME – black). Error bars represent SEM. n = 4 wells each. Dyn = dynorphin, ME = met-enkephalin. (**j**) (Left) representative imaging showing δLight - expressing dissociated hippocampal neurons, scale bar 50 μm. (Right) δLight-expressing dissociated hippocampal neurons respond to ligands in a concentration-dependent manner (ME – green, DynA13 – gray, β-endorphin – black). Error bars represent SEM. n = 4 wells each. Dyn = dynorphin, ME = met-enkephalin, scale bar, 50 μm.

We replaced amino acids between R257 in the intracellular loop (ICL) 3 and R6.24 on the transmembrane domain (TM) 6 of the human κOR, S247 in ICL3 and K6.24 in TM6 of the human δOR, and S6.23 in TM6 and K6.24 in TM6 of human µOR, with a circularly permuted green fluorescent protein (cpGFP), to generate κLight, δLight and µLight sensors, respectively (Fig. 1a,b and Extended Data Fig. 1a,b). The dynamic range of each sensor was optimized by screening linker compositions. In total, the dynamic ranges of 698 κLight variants, 64 δLight variants and 233 µLight variants were examined in response to U50,488, met-enkephalin (ME) and DAMGO, respectively (Extended Data Fig. 1c). To promote excellent membrane localization, we fused a telencephalin (TlcnC) tag^31^ or endoplasmic reticulum (ER) export motif (FCYENEV)^32^ followed by a chain of GS linker and the proximal restriction and clustering (PRC) tag^33^ to the C terminus of κLight, δLight and µLight. We named these new variants κLight1.3, δLight1 and µLight1, respectively. In addition, we mutated D3.22 of κOR and D3.32 in δOR in the binding pockets to attenuate the ligand binding, which led to two control sensors κLight0 and δLight0.

When transiently expressed in mammalian HEK293 cells and dissociated neuronal cultures, we observed excellent membrane expression of κLight1.3, δLight and µLight. All three sensors were activated by their endogenous receptor agonists (100 µM), dynorphin A1-8 (DynA8), ME and β-endorphin, respectively (signal-to-noise ratios (SNR) values for κLight1.3 (HEK) = 7.5 ± 0.45; κLight1.3 (neuron) = 5.6 ± 0.2; δLight (HEK) = 16 ± 0.62; δLight (neuron) = 8.9 ± 0.43; µLight (HEK) = 4.7 ± 0.26) (Fig. 1c,d and Extended Data Fig. 1d). The ligand-induced responses (κLight1.3 change in fluorescence (Δ*F/F*); neuron) = 151% ± 5.1%; δLight Δ*F/F* (neuron) = 123% ± 19.4%; µLight Δ*F/F* (neuron) = 19.6% ± 3.2%) were blocked by naloxone (1 mM), which is an antagonist for all three receptors (Extended Data Fig. 1e).

**Extended Data Fig. 2:**
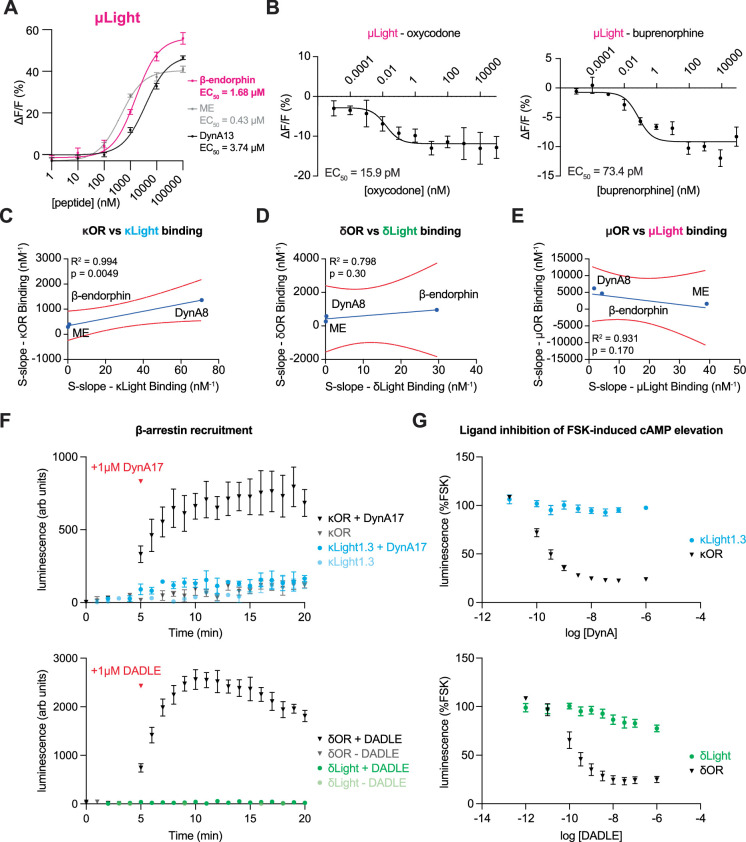
Binding studies of selected compounds on the opioid sensors and receptors. (**a**) Concentration-response curves of µLight-expressing HEK293T cells to three peptide ligands (β-endorphin – magenta, ME – gray, DynA13 – black). Error bars represent SEM. (**b**) Concentration-response curve of µLight-expressing HEK293T cells to oxycodone and buprenorphine. Error bars represent SEM. (**c**) Linear regression plot between the s-slope (maximum displacement / IC50, in nM-1) for κOR (S-slope - κOR Binding) and κLight1.3 (S-slope - κLight Binding) measured with a radio-ligand binding assay. Red curves indicate 95 % confidence interval. (**d**) Linear regression plot between the s-slope (maximum displacement / IC50, in nM-1) for δOR (S-slope - δOR Binding) and δLight (S-slope - δLight Binding) measured from radio-ligand binding assay. Red curves indicate 95 % confidence interval. (**e**) Linear regression plot between the s-slope (maximum displacement / IC50, in nM-1) for µOR (S-slope - µOR Binding) and µLight (S-slope - µLight Binding) measured from radio-ligand binding assay. Red curves indicate 95 % confidence interval. (**f**) NanoBiT assay (κOR / κLight1.3 or δOR / δLight + SmBiT-βarr1 + LgBiT-CAAX) measuring βarrestin1 translocation to plasma membrane upon stimulation with 1 µM DynA17 (top: κOR + DynA17 (black), κOR (gray), κLight1.3 + DynA17 (blue), κLight1.3 (light blue), 1 µM DynA17 added at 5 min) or 1 µM DADLE (bottom: δOR + DADLE (black), δOR (gray), δLight + DADLE (green), δLight (light green), 1 µM DADLE added at 5 min), n = 3 for each experiment, error bars represent SEM. (**g**) GloSensor cAMP assay measuring DynA17 inhibition of forskolin (FSK)-induced cAMP elevation between top: κOR (black) and κLight1.3 (blue), n = 5, and bottom: δOR (black) and δLight (green), n = 4, error bars represent SEM.

To eliminate response variability due to inconsistent expression level of sensors via transient transfection, we developed HEK293T cell lines stably expressing κLight1.3, δLight and µLight. Using these cell lines, we characterized the promiscuity of endogenous opioid peptides on activating sensors^34^. First, all three sensors have consistent excitation peak wavelengths at 495 nm and emission peaks at 515 nm (Extended Data Fig. 1f). Second, in situ titration showed that all three sensors can be activated by three distinct endogenous opioid peptides but with different potency and efficacy. κLight1.3 responded to dynorphin A1-13 (DynA13) with an apparent half maximal effective concentration (EC_50_) of 89.8 pM, which is three magnitudes higher than β-endorphin and ME. However, at higher concentrations (>10 µM), κLight1.3 displayed higher fluorescence changes to β-endorphin, followed by DynA13 and ME (Δ*F/F* (κLight - DynA13) = 93.6% ± 3.9%; Δ*F/F* (κLight - β-endorphin) = 126.9% ± 8.6%; Δ*F/F* (κLight - ME) = 80.3% ± 1.8%; Fig. 1e). δLight was activated by ME with an EC_50_ of 6.5 nM, which is two orders of magnitude greater than DynA13 and β-endorphin, and had higher fluorescence efficacy compared to these two peptides (Δ*F/F* (δLight - DynA13) = 232.6% ± 6.8%; Δ*F/F* (δLight - β-endorphin) = 147.9% ± 4.1%; Δ*F/F* (δLight - ME) = 246.1% ± 4.6%; Fig. 1f). In contrast, we did not observe apparent responses of control sensors when the binding pocket was ablated (κLight0 or δLight0; Extended Data Fig. 1g,h). To further examine the selectivity for κLight1.3 and δLight in the context of neurons, we infected dissociated hippocampal neurons with AAV9-*hSyn*-κLight1.3 and AAV9-*hSyn*-δLight, respectively. We performed in situ titration in dissociated hippocampal neurons in the same context as in the HEK293T stable cell line. Expectably, the selectivity of both sensors in neurons is consistent to that in HEK293T cells (Extended Data Fig. 1i,j). However, all three endogenous opioid peptides showed similar sensor potency and efficacy for µLight activation (Extended Data Fig. 2a), suggesting that further improvement and engineering are required. Together, at presumed physiological conditions (pM–100 nM), both κLight1.3 and δLight are selective and sensitive to endogenous opioid peptides.

Next, we sought to determine the selectivity of antagonists acting on κLight1.3 and δLight. By running the in situ titration in antagonist mode^35^ using the same HEK293T stable cell lines, we were able to determine the selectivity of antagonists acting on κLight1.3 and δLight. In addition to naloxone, we chose nor-binaltorphimine (Nor-BNI), ICI 174864 and CTAP, which selectively antagonize κOR, δOR and µOR, respectively. As expected, increasing the concentration of naloxone (100 nM to 10 µM) shifted the apparent EC_50_ to the right for DynA13 and ME for κLight1.3 and δLight, respectively: naloxone inhibited δLight with twofold greater affinity than κLight (p2A (δLight - naloxone) = 7.64, pA2 (κLight - naloxone) = 5.68). Nor-BNI displayed slightly higher affinity in blocking κLight than δLight (pA2 = 8.28 and 7.3, respectively; Fig. 1g–j,o,p). We did not observe apparent antagonism of κLight by ICI 174864, whereas it effectively inhibited activation of δLight by ME (pA2 δLight - ICI 174864 = 7.17; Fig. 1k,l,o,p). The µOR-selective antagonist CTAP did not affect the EC_50_ of DynA13 or ME in either κLight or δLight, respectively (Fig. 1m–p).

### Selectivity and pharmacology of the opioid biosensors

**Fig. 2:**
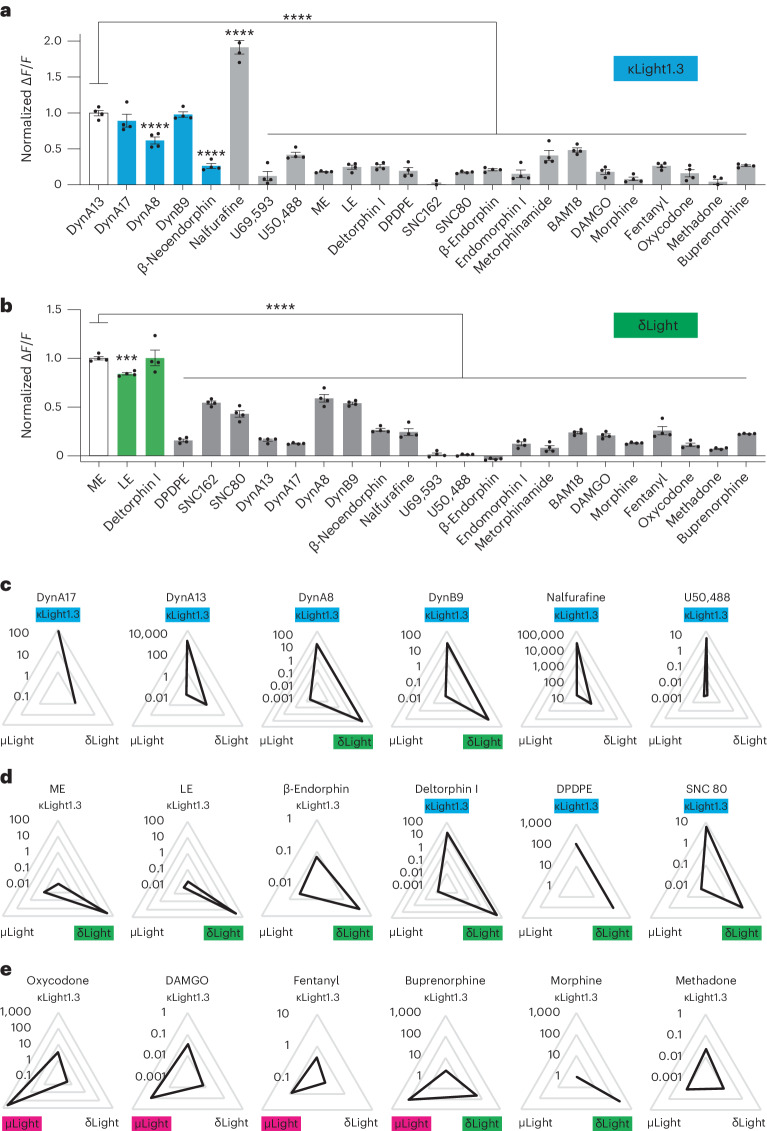
Pharmacological characterization of κLight and δLight. **a**, Normalized Δ*F/F* of κLight1.3 upon addition of the listed compounds (10 nM). Δ*F/F* of all compounds are normalized to DynA13 (DynA13: 1 ± 0.03, DynA17: 0.89 ± 0.08, DynA8: 0.61 ± 0.04, DynB9: 0.97 ± 0.03, β-neoendorphin: 0.26 ± 0.03, nalfurafine: 1.91 ± 0.09, U69,593: 0.12 ± 0.06, U50,488: 0.42 ± 0.03, ME: 0.18 ± 0.005, LE: 0.24 ± 0.02, deltorphin I: 0.26 ± 0.02, DPDPE: 0.19 ± 0.04, SNC162: 0.009 ± 0.02, SNC 80: 0.17 ± 0.008, β-endorphin: 0.21 ± 0.01, endomorphin I: 0.15 ± 0.05, metorphinamide: 0.41 ± 0.06, BAM18: 0.48 ± 0.03, DAMGO: 0.17 ± 0.03, morphine: 0.08 ± 0.02, fentanyl: 0.26 ± 0.02, oxycodone: 0.16 ± 0.04, methadone: 0.04 ± 0.03, buprenorphine: 0.27 ± 0.01; *n* = 4 wells each. ****P < 0.0001, one-way analysis of variance (ANOVA) compared to DynA13 with Sidak’s multiple-comparisons test). Error bars represent the s.e.m. **b**, Normalized Δ*F/F* of δLight upon addition of the listed compounds (10 nM). Δ*F/F* of all compounds were normalized to ME (ME: 1 ± 0.01, LE: 0.84 ± 0.01, deltorphin I: 1 ± 0.07, DPDPE: 0.15 ± 0.01, SNC162: 0.54 ± 0.02, SNC80: 0.42 ± 0.03, DynA13: 0.15 ± 0.01, DynA17: 0.12 ± 0.004, DynA8: 0.58 ± 0.03, DynB1-9: 0.53 ± 0.01, β-neoendorphin: 0.26 ± 0.01, nalfurafine: 0.24 ± 0.03, U69,593: 0.014 ± 0.014, U50,488: 0.009 ± 0.004, β-endorphin: −0.03 ± 0.004, endomorphin I: 0.12 ± 0.01, metorphinamide: 0.07 ± 0.02, BAM18: 0.23 ± 0.01, DAMGO: 0.2 ± 0.01, morphine: 0.12 ± 0.005, fentanyl: 0.25 ± 0.04, oxycodone: 0.11 ± 0.01, methadone: 0.06 ± 0.006, buprenorphine: 0.22 ± 0.003; *n* = 4 wells each. ****P < 0.0001, ***P = 0.0006, one-way ANOVA compared to DynA13 with Dunnett’s multiple-comparisons test). Error bars represent the s.e.m. **c**–**e**, log s-slope values (in nM^−1^) of κOR (**c**), δOR (**d**) and µOR (**e**)-specific ligands plotted in triangle plots (κLight, blue; δLight, green; µLight, magenta). Higher s-slope values are located on the outer triangle. Enk, enkephalin.

We next used a low concentration (10 nM) of a broad panel of endogenous and synthetic ligands to evaluate their rank order of response for inducing sensor fluorescence. We found that known κOR-selective endogenous peptides induced significantly greater fluorescence changes at κLight compared to δOR-selective or µOR-selective ligands. Among the dynorphin peptides, the shorter-form dynorphin DynA8 induced lower activation of κLight compared to DynA13. Interestingly, nalfurafine, a synthetic κOR agonist, elicited an almost twofold greater fluorescence change compared to the dynorphins (Fig. 2a). For δLight cells, enkephalins and δOR-selective agonists elicited larger responses compared to other ligands; deltorphin I displayed similar efficacy as ME and LE for δLight activation (Fig. 2b). Endogenous opioid peptide agonists at µOR, including β-endorphin, endomorphin, metorphinamide and BAM18, displayed various efficacies for κLight1.3 and δLight activation, although to a much smaller extent compared to κOR-specific and δOR-specific peptides. Notably, U50,488 and U69,593 selectively activated κLight over δLight, while SNC80 and SNC162 activated δLight over κLight, confirming the sensors’ specificity to receptor-specific small-molecule agonists (Fig. 2a,b).

We then used radar plots to compare the proportionality constant (s-slope) of various receptor-selective ligands for activating each sensor (Fig. 2c–e and Extended Data Table 1). The s-slope is a constant that links the variables of dynamic range (Δ*F/F*_max_) and EC_50_ of a given sensor response to a drug, defined as Δ*F/F*_max_/EC_50_. It highlights both the efficacy and potency of drugs on sensor responses^36^. By plotting s-slope values of individual ligands on three sensors as a radar plot, we found that the long forms of dynorphin are more potent in activating κLight1.3 than the short forms, the latter of which displayed considerable activity at δLight as well. Both nalfurafine and U50,488 were selective for κLight1.3 (Fig. 2c). The enkephalins (both ME and Leu-Enk (LE)), as well as β-endorphin, were highly selective for δLight, whereas deltorphin I and DPDPE displayed similar s-slopes for κLight1.3 and δLight. Despite low efficacy at κLight1.3, the s-slope of SNC80 was slightly higher at κLight1.3 than that at δLight (Fig. 2d). Notably, µLight was insensitive to morphine, whereas the latter induced slight fluorescence increases at κLight1.3 and δLight. In contrast, methadone activated all three sensors with similar efficacy and potency. Buprenorphine activates all three sensors but showed higher potency for µLight and δLight. On the other hand, other µOR-selective synthetic drugs, including DAMGO, fentanyl and oxycodone, engaged µLight with higher s-slopes compared to κLight1.3 and δLight (Fig. 2e). Interestingly, oxycodone and buprenorphine suppressed, rather than enhanced, µLight fluorescence; thus, the s-slope was calculated using the absolute Δ*F/F*_max_ (Extended Data Fig. 2b).

To determine whether the insertion of cpGFP perturbs the ligand binding properties of these receptor-based opioid sensors, we first assessed the binding profile of both sensors and their corresponding receptors, followed by examining the ability of κLight1.3 and δLight to engage G-protein and β-arrestin pathways coupled to κOR and δOR, respectively. We conducted a radioligand binding assay using cells expressing each sensor and a panel of ligands that includes several endogenous peptides^16,37,38^. For cells stably expressing µLight, endogenous opioid peptides displaced [^3^H] diprenorphine binding with nM IC_50_ except for metorphamide (µM IC_50_). Specific binding detected in the presence of these peptides ranged from 34% ± 2% for peptide F to 82% ± 2% with BAM18. In the case of synthetic agonists, we see that DAMGO and oxycodone have nM IC_50_ while morphine and fentanyl have µM IC_50_. Interestingly, in the case of fentanyl, we found that it exhibits nM IC_50_ in CHO cells stably expressing µORs (Extended Data Table 2). For cells stably expressing δLight, the endogenous opioid peptides and the synthetic agonists displaced [^3^H] diprenorphine binding with nM IC_50_ except for peptide E (µM IC_50_). Specific binding detected in the presence of the endogenous peptides ranged from 32% ± 3% for BAM18 to 77% ± 4% with ME (Extended Data Table 2). For cells stably expressing κLight1.3, the endogenous opioid peptides and the synthetic agonist U69,593 displaced [^3^H] diprenorphine binding with nM IC_50_. Specific binding detected in the presence of the endogenous peptides ranged from 10% ± 5% for ME to 76% ± 2% with DynB13 (Extended Data Table 2). We next compared binding parameters of sensors with those of receptors as previously reported under similar conditions^16^. Using s-slope analysis, we found that binding parameters of sensors and receptors correlated in all three cases (Extended Data Fig. 2c–e). A positive correlation, especially for κLight and δLight, suggested that both radioligand binding assay and fluorescence assay can report peptides’ efficaciousness similarly. Where µLight shows a negative correlation to µOR indicates that the dynamic range and affinity of µLight still needs improvement to reliably report binding profiles for endogenous peptides.

Besides comparing the sensors with radioligand binding, we assessed the signaling conductivity directly on κLight1.3 and δLight. By implementing NanoBiT assay, we measured luminescence values indicating the elevation of β-arrestin1 upon addition of DynA17 comparing between κLight1.3 and κOR, and addition of DADLE comparing between δLight and δOR. Unsurprisingly, the addition of cpGFP eliminated the β-arrestin1-recruiting capability of κLight1.3 and δLight (Extended Data Fig. 2f). On the other hand, we assessed the DynA17 inhibition of forskolin-induced cAMP elevation by applying the GloSensor assay onto κLight1.3 and κOR, and same paradigm for DADLE inhibition onto δLight and δOR. The result indicates that neither κLight1.3 nor δLight is able to reduce the ligand-induced elevation of cAMP signals (Extended Data Fig. 2g).

Together, these data suggest that the cpGFP insertion eliminated the signaling conductivity of the receptors and is not likely to perturb the binding pockets of the parent receptor. Our studies demonstrate that peptide binding to an opioid sensor triggers fluorescence changes that correlate with the binding of the peptide to the receptor, making the sensors serve as useful tools to quantify differences in ligand-driven conformational changes between peptides.

### Imaging dynorphin diffusion in brain tissue with κLight

Photoactivatable or ‘caged’ synthetic variants of opioid NPs or photosensitive nanovesicles can be activated with millisecond precision using short flashes of light and have been optimized for spectrally orthogonal use with GFP-based probes^39^. The spatiotemporal scale over which NP volume transmission occurs in brain tissue has been determined by combining photoactivatable NPs or nanovesicles, electrophysiological recording or cell-based NP biosensors. We thus asked whether κLight can report opioid peptide volume transmission in brain tissue using photo-uncaging experiments.

**Fig. 3:**
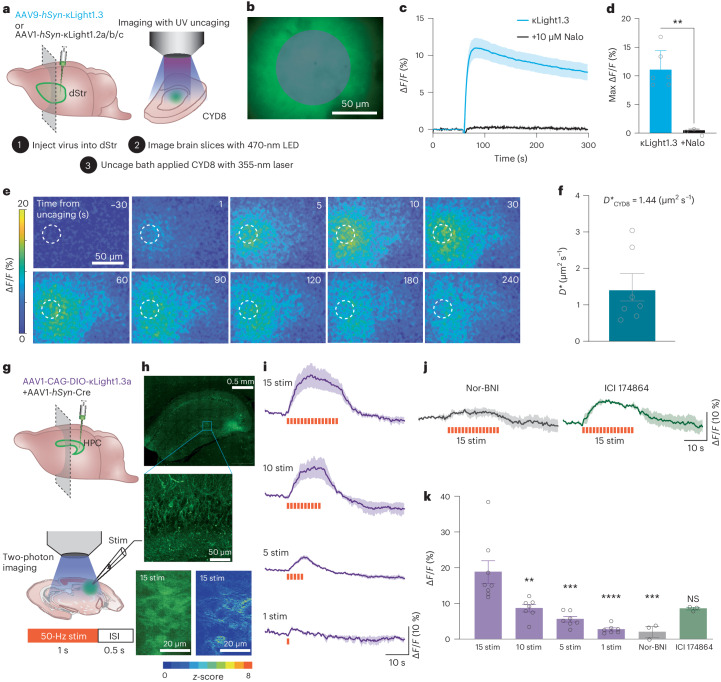
κLight1.3 characterization in acute brain slices. **a**, Schematics shows imaging of striatal acute brain slices and photo-uncaging CYD8 with a 355-nm laser. **b**, Time-lapse images (semitransparent gray circle shows the field of UV illumination). Scale bar, 50 μm. **c**, Response of κLight1.3 to CYD8 photo-uncaging (blue, *n* = 6 slices) in the absence and presence of naloxone (Nalo, 10 μM; black, *n* = 3 slices). Solid lines represent the mean, and the shaded areas represent the s.e.m. **d**, Quantification of the peak Δ*F/F* evoked by CYD8 photo-uncaging. κLight1.3 (blue); 11.1% ± 1.36%, *n* = 6, + naloxone (black); 0.51% ± 0.12%, *n* = 3, *P* = 0.0011, two-tailed unpaired *t*-test. **e**, Time course of κLight1.2a after CYD8 (5 μM) photo-uncaging in the dStr. The dashed circle indicates the site of UV illumination. Heat map indicates Δ*F/F* (%). Scale bar, 50 µm. **f**, Summary of experiments determining the apparent diffusion coefficient, *n* = 7 slices from 4 mice. D* = 1.439 ± 0.37 μm^2^ s^−1^. **g**, Schematics show local electrical stimulation of hippocampal slice with trains of 1-s, 50-Hz stimuli with a 0.5-s interstimulus interval. HPC, hippocampus; ISI, interstimulation interval. **h**, Representative image showing expression of κLight1.3a in CA3 (top; scale bar, 0.5 mm) and zoomed in to visualize the localization of localization κLight1.3a to the membranes of neuronal processes in the dentate gyrus (middle; scale bar, 50 μm). Representative two-photon field of view from 15 stimulations (bottom) indicating the averaged intensity across all frames and *z*-score of responses in the representative field of view; scale bar, 20 μm. **i**,**j**, Average κLight1.3a responses to various electrical stimulation in the absence (**i**) and presence (**j**) of antagonists, Nor-BNI (gray) and ICI 174864 (green). Solid lines represent the mean, and shaded areas represent the s.e.m. **k**, Bar graph summarizing the peak fluorescence response to each stimulation condition. 15 stim (*n* = 8 slices): 14.3% ± 2.4%, 10 stim (*n* = 7 slices): 6.62% ± 0.8%, 5 stim (*n* = 7 slices): 4.28% ± 0.6%, 1 stim (*n* = 7 slices): 2.12% ± 0.3%, Nor-BNI (100 µM, *n* = 3 slices): 1.57% ± 1.2%, ICI 174864 (100 µM, *n* = 3 slices): 6.44% ± 0.3%. Ordinary one-way ANOVA with Bonferroni’s multiple-comparisons test, individual conditions compared to 15 stim, 15 stim versus 10 stim: **P = 0.044, 15 stim versus 5 stim: ***P = 0.0001, 15 stim versus 1 stim: ****P < 0.0001, 15 stim versus Nor-BNI: ***P = 0.0002, 15 stim versus ICI 174864: not significant (NS), *P* = 0.0525. Error bars represent the s.e.m.

**Extended Data Fig. 3:**
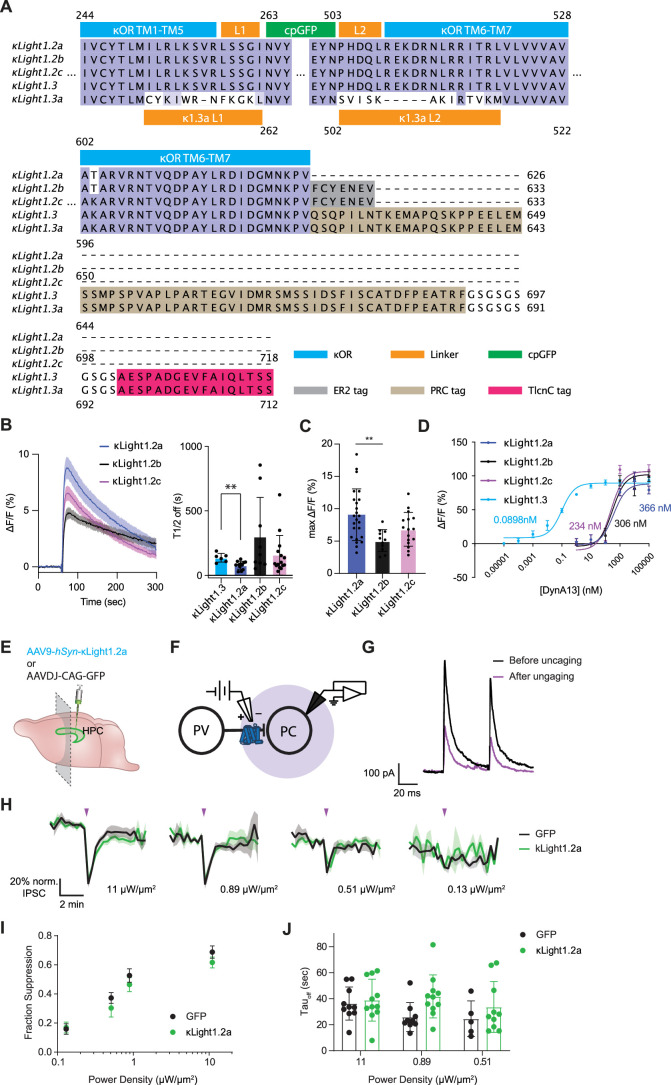
Comparison of top κLight variants and buffering effect study. (**a**) Sequence alignment of κLight1.2a, κLight1.2b, κLight1.2c, κLight1.3, and κLight1.3a. Purple indicates the same residues across all variants. Blank indicates different residues. Blue color indicates κOR sequence. Orange indicates linkers. Green indicates cpGFP sequences. Gray indicates the sequence for the ER2 tag. Khaki indicates the sequence for the PRC tag, and magenta indicates the TlcnC tag. (**b**) (left) CYD8 uncaging response comparison between κLight1.2a (dark blue, n = 12 videos), κLight1.2b (black n = 9 videos), and κLight1.2c (magenta, n = 15 videos) expressed in dStr in acute brain slices. The solid line represents the mean, and the shaded area represents SEM. (right) κLight1.2a showed faster T1/2 off dynamics compared to κLight1.3 (κLight1.3 T1/2 extracted from Fig. 3c, 137 ± 14, n = 6; κLight1.2a, 79 ± 8.9, n = 12; κLight1.2b, 301 ± 101, n = 9; κLight1.2c, 156 ± 40, n = 15, **p = 0.001, one-way ANOVA). Error bars represent SEM. (**c**) Max ΔF/F (%) at the peak of the CYD8 uncaging responses for κLight1.2a (blue, n = 24 videos), κLight1.2b (black, n = 11 videos), and κLight1.2c (magenta, n = 16 videos). κLight1.2a: 9.09 ± 0.8 %, κLight1.2b: 5.1 ± 0.5 %, κLight1.2c: 6.84 ± 0.7 %. **p = 0.0027, ordinary one-way ANOVA with Dunnett’s multiple comparisons test. Error bars represent SEM. (**d**) Dose response curves for DynA13 at κLight1.2a (dark blue, n = 3 wells, EC50 = 366 nM), κLight1.2b (black, n = 5 wells, EC50 = 306 nM), κLight1.2c (magenta, n = 4 wells, EC50 = 234 nM), and reused κLight1.3 trace from Fig. 1e (blue, n = 4 wells, 0.0898 nM). Error bars represent SEM. (**e**) Schematic indicating injection of C57/B6J pups with AAV1-*hSyn*-κLight1.2a or AAV-DJ-CAG-GFP in the hippocampus followed by 3 weeks of expression prior to preparation of acute brain slices for electrophysiology. (**f**) Schematic of the electrophysiological recording configuration. Whole-cell voltage-clamp recordings are obtained from pyramidal cells (PCs) held at 0 mV while parvalbumin (PV) basket cell axons are preferentially stimulated with a narrow-tipped theta-glass-based bipolar stimulating electrode. Two electrical stimuli are applied 50 ms apart to drive synaptic inhibition. A 5 ms flash of 355 nm light (semitransparent purple circle) is applied to photorelease DynA8, which acts on presynaptic mu and delta opioid receptors on the PV cell to suppress the synaptic output. (**g**) Example inhibitory post-synaptic currents (IPSCs) before (black) and after (purple) DynA8 photorelease. (**h**) Time-course of IPSC suppression in response to DynA8 photorelease in slices expressing κLight1.2a (n = 13 cells from 3 mice, green) or GFP (n = 10 cells from 3 mice, black) using different intensities of UV light. Traces indicate the mean peak IPSC (normalized to 100%) over time, which was probed every 20 sec. Purple arrows indicate the application of UV light. The solid lines represent the mean, and the shaded areas represent SEM. (**i**) Power-response curve summarizing the fraction of the baseline IPSC suppressed by DynA8 photorelease in slices expressing κLight1.2a (n = 13 cells from 3 mice, green) or GFP (n = 10 cells from 3 mice, black). No significant differences were detected at different power densities (Two tailed multiple Mann-Whitney tests between GFP and κLight1.2a at different power densities: 0.13 µW/µm2: p = 0.91, 0.51 µW/µm2: p = 0.71, 0.89 µW/µm2: p = 0.71, 11 µW/µm2: p = 0.70, non-significant). (**j**) Average time constant of IPSC recovery after DynA8 photorelease in slices expressing κLight1.2a (n = 3 mice, green) or GFP (n = 3 mice, black). No significant differences were detected (Two tailed multiple Mann-Whitney tests between GFP and κLight1.2a at different power densities: 0.51 µW/µm2: κLight1.2a n = 10 cells, GFP n = 5 cells, p = 0.52; 0.89 µW/µm2: κLight1.2a n = 11 cells, GFP n = 9 cells, p = 0.04; 11 µW/µm2: κLight1.2a n = 11 cells, GFP n = 10 cells, p = 0.56, non-significant). Error bars represent SEM.

To choose the most appropriate κLight variant that balances dynamic range and sensitivity, we first examined the responses and kinetics of various κLight variants using photoactivable DynA8 (CYD8)^29^. We injected AAV9-*hSyn*-κLight1.x (top κLight variants including 1.2a, 1.2b, 1.2c and 1.3) into the dorsal striatum (dStr) of C57 mouse pups (postnatal day (P)0–P3) and prepared the brain slices after 3 weeks of expression (Fig. 3a). On the day of imaging, CYD8 was circulated in the bath and photo-uncaged with a 50-ms flash of 355-nm laser light over an area of 3,800 µm^2^, while imaging the responses of κLight with a 473-nm LED within the same region (Fig. 3b). Among all the κLight variants tested (Extended Data Fig. 3a), κLight1.3 yielded the greatest response (Δ*F/F* = 11% ± 1.4%; Fig. 3c,d), followed by κLight1.2a (Δ*F/F* = 9.09% ± 0.81%), κLight1.2c (Δ*F/F* = 6.84% ± 0.65%) and κLight1.2b (Δ*F/F* = 5.1% ± 0.51%; Extended Data Fig. 3b,c). The uncaging response was completely blocked by the presence of naloxone (0.5% ± 0.1%; Fig. 3d), confirming that the fluorescence change is due to ligand-dependent sensor activation, as opposed to being an artifact of the ultraviolet (UV) light flash. While κLight1.3 had the greatest Δ*F/F*, we noticed that its response was slow to decay in comparison to κLight1.2a (tau_off_ - κLight1.3 = 202.1 s, tau_off_ - κLight1.2a = 179.7 s, tau_off_ - κLight1.2b = 246.1 s, tau_off_ - κLight1.2c = 165.0 s; Fig. 3c and Extended Data Fig. 3b), presumably due to the higher affinity for dynorphins that results in slower peptide dissociation (Extended Data Fig. 3d).

We next examined whether sensor expression might alter the ability of peptide ligands to engage endogenous opioid receptors. For this experiment, we used κLight1.2a, which exhibited faster decay kinetics than κLight1.3 upon DynA8 photorelease (Extended Data Fig. 3b), yet still produced a relatively large Δ*F/F*. Adeno-associated viruses (AAVs) encoding κLight1.2a or GFP control were injected into the hippocampus of C57 pups (P0–P3) and allowed to express for a minimum of 3 weeks before acute slices were prepared for electrophysiology (Extended Data Fig. 3e). Parvalbumin interneurons in the CA1 region of the hippocampus express µOR and δOR, which act presynaptically to suppress synaptic transmission^40^. Although DynA8’s primary target is κOR, it also binds to µOR and δOR (for example, Fig. 2b and Extended Data Table 1)^41^. This allowed us to ask whether the activation of µOR and δOR by DynA8 is altered by the expression of κLight1.2a. To assay opioid receptor function, we recorded inhibitory postsynaptic currents (IPSCs) in pyramidal cells, evoked with a stimulation protocol that favors µOR-sensitive and δOR-sensitive parvalbumin synapses^40^ (Extended Data Fig. 3f). Photorelease of DynA8 using 5-ms flashes of 355-nm light produced a rapid, power-dependent reduction in IPSC amplitude that reversed over the course of several minutes (Extended Data Fig. 3g,h). Compared to GFP control, κLight1.2a expression altered neither the degree of IPSC suppression, nor the time course of IPSC recovery in response to DynA8 photorelease across all light power densities examined (Extended Data Fig. 3i,j). These results suggest that κLight1.2a expression does not result in sufficient ligand buffering as to perturb the activation of endogenous opioid receptors.

**Extended Data Fig. 4:**
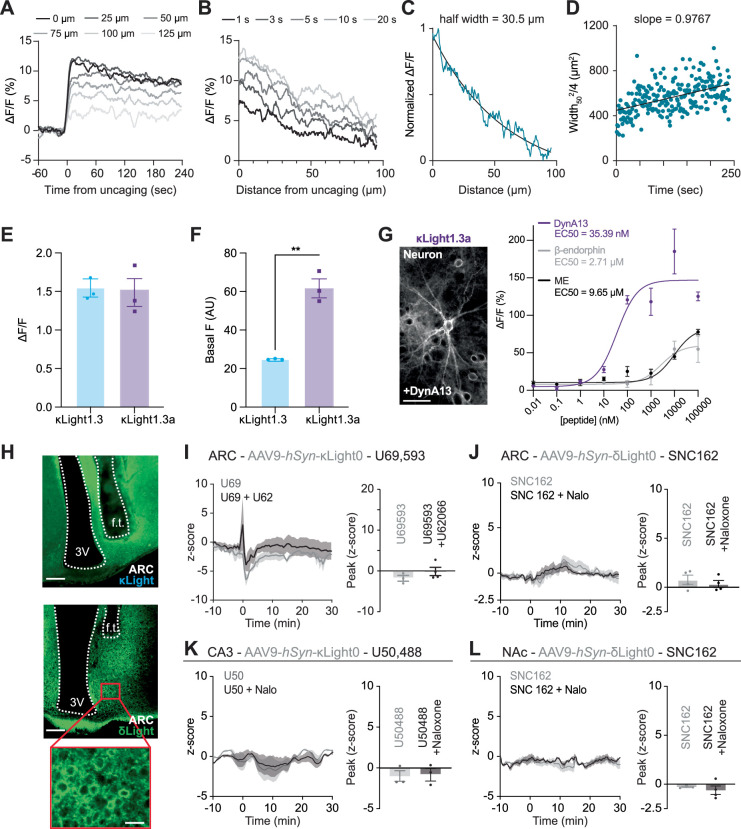
Dynorphin diffusion analysis and in vivo pharmacology with control sensors. (**a**) Representative example of the fluorescence response of κLight1.2a for single pixels along the center of the imaging field at various distances from the site of DynA8 photorelease. (**b**) Representative examples of fluorescence profile as a function of distance from the uncaging site at differing time points after uncaging. (**c**) Representative example of a fluorescence profile at a single time (5 sec, as in Extended Data Fig. 4b), normalized and fit to an exponential function in order to extract the half width (30.5 µm). (**d**) Representative plot of the half-width squared/4 vs. time for determination of the apparent diffusion coefficient D*. The fluorescence profile fits (for example Extended Data Fig. 4c) were repeated in 1 second time bins to extract the half-width. The slope of this linear regression is the apparent diffusion coefficient D*. (**e**) Fluorescence response (ΔF/F) to U50,488 (100 μM) compared between κLight1.3 (blue, n = 3 wells), and κLight1.3a (magenta, n = 3 wells). κLight1.3: 1.56 ± 0.12, κLight1.3a: 1.474 ± 0.18, two tailed unpaired t test, p = 0.724, non-significant. Error bar represents SEM. (**f**) Basal fluorescence compared between κLight1.3 (blue, n = 3 wells), and κLight1.3a (magenta, n = 3 wells). κLight1.3: 25.0 ± 0.08, κLight1.3a: 61.01 ± 4.49, two tailed unpaired t test, **p = 0.0013. Error bar represents SEM. (**g**) (Left) representative imaging showing κLight1.3a - expressing dissociated hippocampal neurons, scale bar 50 μm. (Right) κLight1.3a-expressing dissociated hippocampal neurons respond to ligands in a concentration-dependent manner (DynA13 – magenta, β-endorphin – gray, ME – black). Error bars represent SEM. n = 4 wells each. Dyn = dynorphin, ME = met-enkephalin. (**h**) Representative images showing κLight (top), δLight (middle), and zoomed-in image for δLight (bottom) expression in ARC. Scale bar 150 µm for both κLight and δLight. δLight zoomed insert has scale bar = 30 µm. Abbreviations: ventricle (3 V), fiber track (f.t.). (**i**) (Left) κLight0 response to 3 mg/kg U69,593 (gray, n = 3 animals), and 3 mg/kg U69,593 + 1 mg/kg U62,066 (black, n = 4 animals) in ARC, Solid lines represent the mean, and shaded areas represent SEM. (Right) bar graph indicating the peak z-score of each response, U69,593: -1.9 ± 0.7 %, U69,593 + U62,066: −0.2 ± 1 %, unpaired t test, p = 0.2625, non-significant. U69 = U69,593, U62 = U62,066. Error bars represent SEM. (**j**) (Left) δLight0 response to 5 mg/kg SNC162 (gray, n = 4 animals), and 5 mg/kg SNC162 + 4 mg/kg naloxone (black, n = 4 animals) in ARC. Solid lines represent the mean, and shaded areas represent SEM. (Right) bar graph indicating the peak z-score of each response, SNC162: 0.77 ± 0.5 %, SNC162 + naloxone: 0.33 ± 0.4 %, unpaired t test, p = 0.4948, non-significant. Nalo = Naloxone. Error bars represent SEM. (**k**) (Left) κLight0 response to 10 mg/kg U50,488 (gray, n = 3 animals), and 10 mg/kg U50,488 + 10 mg/kg naloxone (black, n = 3 animals) in CA3. Solid lines represent the mean, and shaded areas represent SEM. (Right) bar graph indicating the peak z-score of each response, U50,488: −1 ± 0.7 %, U50,488 + naloxone: −0.75 ± 0.8 %, two tailed unpaired t test, p = 0.8123, non-significant. U50 = U50,488, Nalo = Naloxone. Error bars represent SEM. (l) (Left) δLight0 response to 5 mg/kg SNC162 (gray, n = 4 animals), and 5 mg/kg SNC162 + 4 mg/kg naloxone (black, n = 3 animals) in ARC, Solid lines represent the mean, and shaded areas represent SEM. (Right) bar graph indicating the peak z-score of each response, SNC162: −0.61 ± 0.4 %, SNC162 + naloxone: −0.27 ± 0.07 %, two tailed unpaired t test, p = 0.5451, non-significant. Nalo = Naloxone. Error bars represent SEM.

We next measured the spread of DynA8 in space and time. AAV1-*hSyn*-κLight1.2a was injected into dStr and imaging was performed 3 weeks after injection (Fig. 3a). Small volumes of DynA8 were rapidly photoreleased using a focused 25-µm-diameter spot of 355-nm light (Fig. 3e) while monitoring sensor activation at distances of up to 125 µm away. We observed that the peak Δ*F/F* decreased with increased time from uncaging and with distance from the uncaging site (Extended Data Fig. 4a). For each video frame after uncaging, we plotted the fluorescence profile as a function of distance from the uncaging spot and extracted the Δ*F/F* half-width, which was used to compute an effective diffusion coefficient (D*) of 1.4 ± 0.4 µm^2^ s^−1^ (*n* = 7 slices from four mice) for DynA8 in dStr (Extended Data Fig. 4b–d). These results suggest that DynA8 can reach receptors over 100 µm away from release sites within several seconds of release in the dStr.

### Two-photon imaging of dynorphin release via electrical stimulation

It has been historically difficult to determine the electrical parameters that can effectively trigger the release of endogenous opioid peptides in brain tissue. We thus examined if κLight can detect endogenous opioid peptide release triggered by electrical stimulation ex vivo. To do so, we first improved the basal fluorescence of κLight1.3 by integrating CYKIWRNFKGK as linker 1 and SVISKAKIRTV as linker 2 derived from the oxytocin sensor MTRIA_OT_^42^ (Extended Data Fig. 3a). This new variant, named κLight1.3a, displayed a similar dynamic range (κLight1.3 at 155% ± 11.6%, κLight1.3a at 152% ± 29.5%, *P* = 0.92, unpaired *t*-test), but >2× the basal brightness compared to κLight1.3 (κLight1.3 at 25 ± 0.08, κLight1.3a at 61.8 ± 7.6, *P* = 0.0075, unpaired *t*-test; Extended Data Fig. 4e,f). To validate that κLight1.3a retain the same selectivity, we performed in situ titration in dissociated hippocampal neuronal cultures using peptides DynA13, ME and β-endorphin. As expected, κLight1.3a showed high selectivity to DynA13 over the other two endogenous peptides (Extended Data Fig. 4g). Immunoreactivity studies have shown abundant dynorphin stored in dentate granule cells, dynorphin dynamics in CA3 have also been shown to have an association with stress under various behavior paradigms, and dynorphins have been shown to inhibit excitatory neurotransmission and prevent the induction of long-term potentiation in hippocampus^43–45^. We sparsely expressed κLight1.3a in CA3 by delivering AAV1-CAG-DIO-κLight1.3a in combination with AAV1-*hSyn*-Cre (Fig. 3g). After 3 weeks of expression, we observed bright labeling of neurons in CA3 and dentate gyrus with clear processes in the basal state and distribution of responses in the field of view using two-photon imaging (Fig. 3h).

Next, we evaluated the responses of κLight1.3a to a range of electrical stimuli parameters applied locally via a stimulating electrode in CA3. Trains of electrical stimuli (1 s at 50 Hz, 0.5-s interstimulus interval) produced sustained fluorescence increases that rapidly decayed upon cessation of the stimulus (Fig. 3i), with an increasing number of stimuli driving larger maximum fluorescence responses (15 stimulations: 14.3% ± 2.4%, 10 stimulations: 8.39% ± 1.9%, 5 stimulations: 4.28% ± 0.6%, 1 stimulation: 2.12% ± 3.3%; Fig. 3k).

The response to 15 stimuli was strongly attenuated by the addition of the κOR antagonist nor-BNI (100 μM, ∆*F/F* = 1.57% ± 1.2%), consistent with the observed fluorescence increase resulting from activation by endogenous peptide. In the presence of δOR antagonist ICI 174864 (100 μM), the responses were decreased but not statistically significant (Fig. 3j,k; ∆*F/F* = 6.44% ± 0.3%).

### Probing the effect of receptor-selective opioid ligands in vivo

**Fig. 4:**
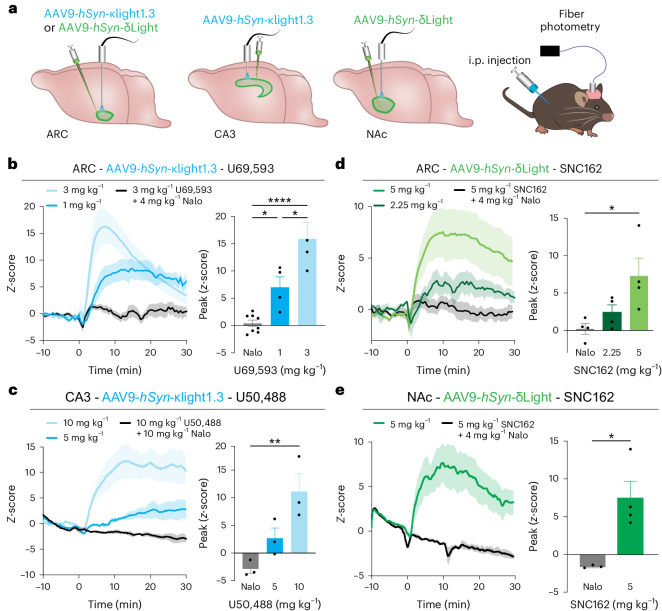
In vivo drug pharmacology imaged with κLight and δLight. **a**, Experimental schematics of κLight1.3 and δLight injection in the hypothalamus (ARC), the hippocampal CA3 and the NAc, followed by imaging with fiber photometry during drug injection. **b**, κLight1.3 response in ARC to different doses of U69,593, 3 mg per kg body weight (light blue), 1 mg per kg body weight (blue) and 3 mg per kg body weight U69,593 + 4 mg per kg body weight naloxone (black); *n* = 7 animals. Solid lines represent the mean, and the shaded area represents the s.e.m. Bar graph indicating the peak *z*-score of each response, 3 mg per kg body weight + naloxone: 0.4% ± 0.6%, 1 mg per kg body weight: 7.0% ± 1.9%, 3 mg per kg body weight: 15.9% ± 3.1%, ordinary one-way ANOVA with Tukey’s multiple-comparisons test, 1 versus 3 *P = 0.012, 1 versus Nalo *P = 0.029, 3 versus Nalo ****P < 0.0001. **c**, κLight1.3 response to different doses of U50,488 in CA3, 10 mg per kg body weight (light blue), 5 mg per kg body weight (blue) and 10 mg per kg body weight U50,488 + 10 mg per kg body weight naloxone (black) in CA3; *n* = 3 animals. Solid lines represent the mean, and shaded areas represent the s.e.m. Bar graph indicating the peak z-score of each response, 10 mg per kg body weight + naloxone: −2.9% ± 0.8%, 5 mg per kg body weight: 2.7% ± 1.8%, 10 mg per kg body weight: 11.1% ± 3.2%, ordinary one-way ANOVA with Dunnett’s multiple-comparisons test, **P = 0.0072. **d**,**e**, δLight response to different doses of SNC162 in ARC (**d**) and NAc (**e**), 5 mg per kg body weight (light green), 2.25 mg per kg body weight (green) and 5 mg per kg body weight SNC162 + 4 mg per kg body weight naloxone (black) in ARC and NAc; *n* = 4 animals. Solid lines represent the mean, and shaded areas represent the s.e.m. Bar graph indicating the peak *z*-score of each response; in ARC: 0.2% ± 0.7%, 2.25 mg per kg body weight: 2.4% ± 1.0%, 5 mg per kg body weight: 7.3% ± 2.4%, ordinary one-way ANOVA with Tukey’s multiple-comparisons test, *P = 0.0258; in NAc: 1.7 ± 0.1%, 5 mg per kg body weight: 7.5% ± 2.2%; two-tailed unpaired *t*-test, *P = 0.0185. In **b**–**e**, error bars represent the s.e.m.

We next determined if κLight and δLight can be activated by systemic administration of exogenous small-molecule drugs in vivo. We injected AAV9-*hSyn* encoding κLight1.3 or δLight, and κLight0 or δLight0 in the arcuate nucleus (ARC) of the hypothalamus^46^, hippocampal CA3 region^43^ and NAc^30^, areas abundant in κOR and δOR. We next implanted fiber-optic ferrules above each injection site and recorded the fluorescence of κLight and δLight upon intraperitoneal (i.p.) injection of opioid receptor-selective ligands using fiber photometry (Fig. 4a and Extended Data Fig. 4h–j).

In each case, we observed dose-dependent fluorescence increases in response to systemic drug i.p. treatment, which were blocked by the nonselective opioid receptor antagonist naloxone. In the ARC, κLight1.3 responded to the κOR-selective agonist U69,593 with a robust increase in fluorescence within a few minutes of drug injection (1 mg per kg body weight: *z*-score_peak_ = 7.0 ± 1.9, 3 mg per kg body weight: *z*-score_peak_ = 15.9 ± 3.05). Co-injection of naloxone (4 mg per kg body weight) drastically attenuated the response to U69,593 (3 mg per kg body weight; U69,593 + naloxone *z*-score_peak_ = 0.39 ± 0.59; Fig. 4b). In CA3, the κOR-selective agonist U50,488 similarly activated κLight1.3 in a dose-dependent manner. Again, the response to U50,488 (10 mg per kg body weight) was completely blocked by co-injecting naloxone (10 mg per kg body weight; 5 mg per kg body weight: *z*-score_peak_ = 2.68 ± 1.8; 10 mg per kg body weight: z-score_peak_ = 11.1 ± 3.2; U50,488 + naloxone: *z*-score_peak_ = −2.86 ± 0.83; Fig. 4c).

In the ARC, SNC162 administration produced increases in δLight fluorescence (2.25 mg per kg body weight: *z*-score_peak_ = 2.4 ± 1.0; 5 mg per kg body weight: z-score_peak_ = 7.28 ± 2.4) that were blocked by naloxone (4 mg per kg body weight) co-injected with SNC162 (5 mg per kg body weight; SNC162 + naloxone: *z*-score_peak_ = 0.19 ± 0.72; Fig. 4d). In the NAc, the administration of SNC162 (5 mg per kg body weight) also increased δLight fluorescence (SNC162: *z*-score_peak_ = 7.45 ± 2.20), and this was again blocked by naloxone (4 mg per kg body weight; SNC162 + naloxone: *z*-score_peak_ = −1.66 ± 0.11; Fig. 4e).

Importantly, we did not observe fluorescence changes in response to agonist when the non-functional mutant sensors κLight0 or δLight0 were expressed in the ARC, CA3 and NAc (Extended Data Fig. 4k,l). These results suggest that both sensors can be faithfully activated by receptor-specific agonists in vivo and ensure a good dynamic range, adequate expression and fiber-expression alignments as a foundation for the following optogenetic and behavioral experiments.

### Measuring dynorphin release via circuit-specific photostimulation

Although optogenetics has been broadly used to trigger neuromodulator release and neural activity, direct monitoring of peptide release triggered by optogenetic stimulation in vivo, especially in a circuit-specific manner with high temporal resolution, has not been measured optically. NAc contains abundant dynorphin, and previous studies have demonstrated that targeting the Dyn-κOR system in the nucleus accumbens shell (NAcSh) can modulate both rewarding and aversive behaviors^47,48^. Furthermore, previous work has demonstrated the ability to measure the optogenetically evoked release of dynorphin in the NAcSh using in vivo opto-dialysis^30^. Studies have also shown that the basolateral amygdala (BLA) sends dense, functional excitatory projections to the NAcSh and that these terminals are sensitive to modulation by Dyn-κOR^49,50^. We, therefore, set out to determine if κLight can detect photostimulated release in vivo in BLA to NAcSh projections.

**Fig. 5:**
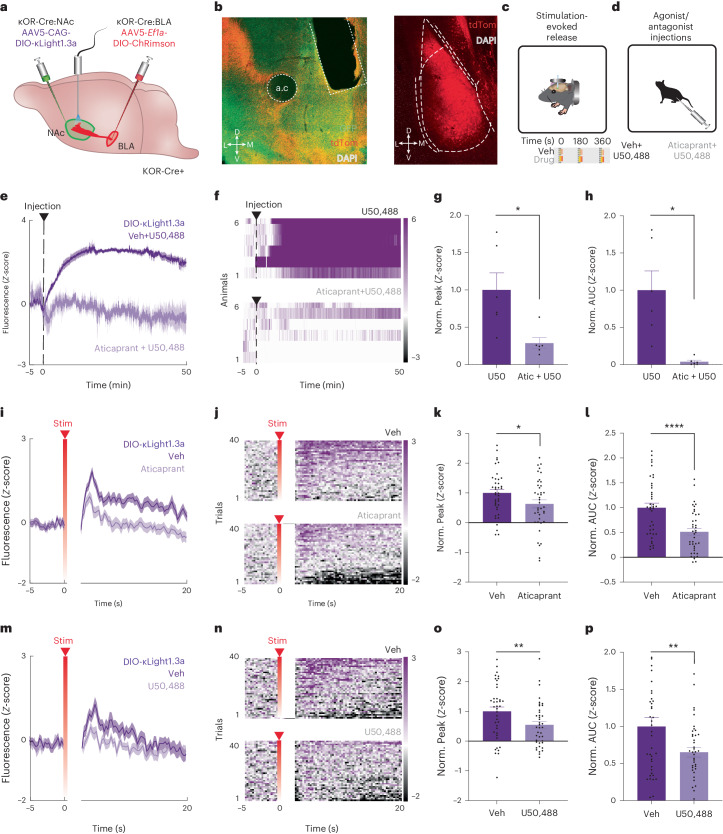
Imaging optogenetically stimulated dynorphin release with κLight1.3a. **a**, Schematic showing κLight1.3a-expressed NAcSh and ChRimson into the BLA of κOR-Cre^+^ mice. **b**, Representative ×20 coronal image (left) showing expression of κLight1.3a (green), ChRimson (red), DAPI (white) and fiber placement in the NAcSh (left; scale bar, 200 μm), and ChRimson (red) and DAPI (white) in the BLA (right; scale bar, 200 μm) from six animals, which showed similar results. **c**,**d**, Schematic of in vivo head-fixed stimulation-evoked dynorphin release (stimuli occurred at 0, 180 and 360 s; **c**) and agonist/antagonist drug injection (10 mg per kg body weight U50,488 and 3 mg per kg body weight aticaprant + 10 mg per kg body weight U50,488; **d**) experiments. **e**,**f**, Mean (**e**) and heat map (**f**) of κLight1.3a activity either averaged across all animals (**e**) or from individuals (**f**) following i.p. injections of vehicle (veh) + U50,488 (dark) and aticaprant + U50,488 (light; *n* = 6 animals). Solid lines represent the mean, and shaded areas represent the s.e.m. **g**,**h**, Normalized peak fluorescence (**g**) and AUC (**h**) of single trials during the injection period (0–50 min; U50: 1 ± 0.23, Atic + U50: 0.29 ± 0.07; two-tailed paired *t*-test, *P = 0.034, *P = 0.014, *n* = 6 animals). Data are represented as the mean ± s.e.m. Atic, aticaprant; U50, U50,488. **i**, Mean κLight1.3a activity averaged across all trials following vehicle (dark) and aticaprant (light) treatment during ChRimson stimulation-evoked trials (*n* = 4 animals). Solid lines represent the mean, and shaded areas represent the s.e.m. **j**, Heat map raster plot of κLight1.3a activity averaged across all trials following vehicle (top) and aticaprant (bottom) treatment during ChRimson stimulation-evoked trials (*n* = 4 animals) displayed in ascending trial order by average activity across trials. ‘Stim’ indicates the time of stimulus application. **k**,**l**, Normalized peak fluorescence (**k**) and AUC of single trials (**l**) across vehicle and aticaprant treatment during all ChRimson stimulation-evoked trials (0–20 s; veh: 1 ± 0.12, Atic: 0.63 ± 0.14; two-tailed paired *t*-test, *P = 0.037, ****P < 0.000, *n* = 4 animals). Data are represented as the mean ± s.e.m. **m**,**n**, Mean (**m**) and heat map raster plot (**n**) of recorded κLight1.3a activity averaged across all trials following vehicle (dark) and U50,488 (light) treatment during ChRimson stimulation-evoked trials (*n* = 4 animals). Solid lines represent the mean, and shaded areas represent the s.e.m. **o**,**p**, Normalized peak fluorescence (**o**) and AUC of single trials (**p**) across vehicle and U50,488 treatment during all ChRimson stimulation-evoked trials (0–20 s; veh: 1 ± 0.15, U50: 0.55 ± 0.12; two-tailed paired *t*-test, **P = 0.002, **P = 0.007, *n* = 4 animals). Data are represented as the mean ± s.e.m.

**Extended Data Fig. 5:**
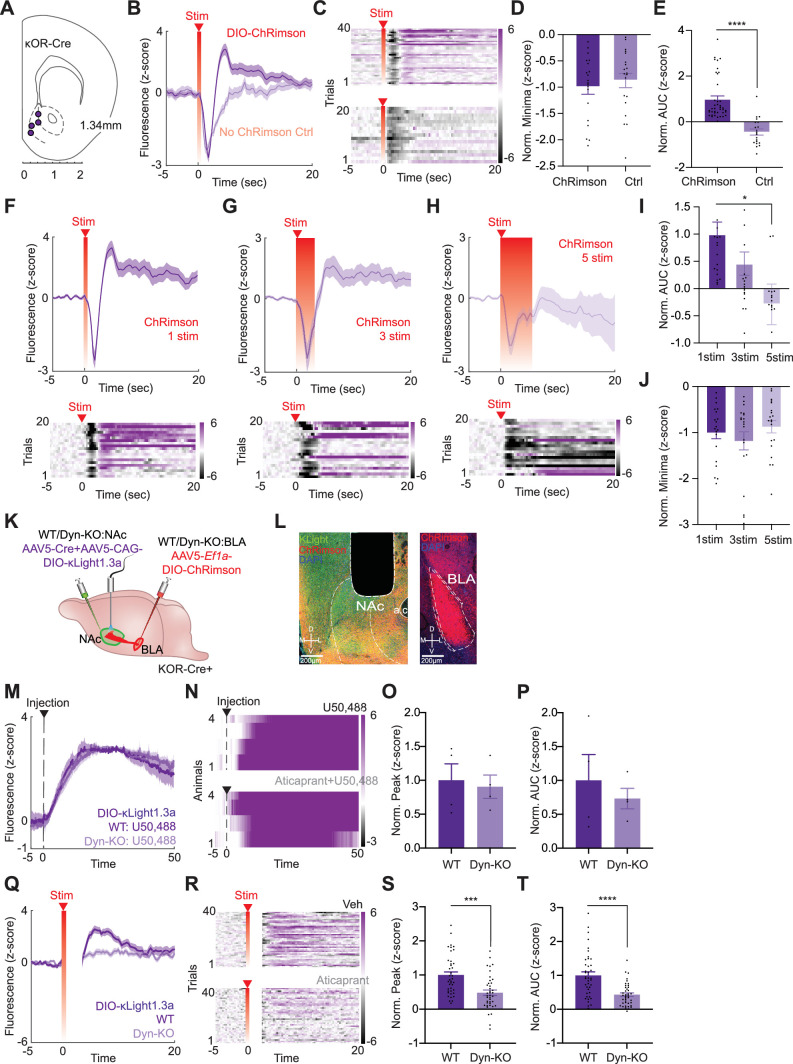
Controls for in vivo fiber photometry with optogenetics and other stimulation parameters. (**a**) Coronal brain schematic showing photometry fiber placements in the NAcSh of κOR-Cre+ mice. (**b**) Mean recorded κLight1.3a fluorescence averaged across all stimulation-evoked trials in ChRimson-injected (dark purple; n = 4 mice) or control mice (light purple; n = 2 mice). Solid lines represent the mean, and shaded areas represent SEM. (**c**) Heatmap raster plot of recorded κLight1.3a fluorescence averaged across all stimulation-evoked trials in ChRimson-injected (top; n = 4 mice) or control mice (bottom; n = 2 mice). (**d**) Normalized fluorescence minima during all stimulation-evoked trials. (0-20 sec; ChRimson – n = 4 animals: −1 ± 0.14, Control - n = 2 animals: -0.87 ± 0.14, two tailed unpaired t test, p = 0.51, non-significant). Data represented as mean ± SEM. (**e**) Normalized fluorescence area under the curve of single trails during all stimulation-evoked trials (0-20 sec; ChRimson – n = 4 animals: 0.98 ± 0.15, Control - n = 2 animals: −0.45 ± 0.13, two tailed unpaired t test, ****p < 0.0001). Data represented as mean ± SEM. (**f**) Mean trace and heatmap raster plot of recorded κLight1.3a fluorescence averaged across all 1 second stimulation-evoked trials in ChRimson-injected mice (n = 4 animals). Stim indicates the time of stimulus application. Solid lines represent the mean, and shaded areas represent SEM. (**g**) Mean trace and heatmap raster plot of recorded κLight1.3a fluorescence averaged across all 3 second stimulation-evoked trials in ChRimson-injected mice (n = 4 animals). Stim indicates the time of stimulus application. Solid lines represent the mean, and shaded areas represent SEM. (**h**) Mean trace and heatmap raster plot of recorded κLight1.3a fluorescence averaged across all 5 second stimulation-evoked trials in ChRimson-injected mice (n = 4 animals). Stim indicates the time of stimulus application. Solid lines represent the mean, and shaded areas represent SEM. (**i**) Normalized fluorescence area under the curve of single trails during all stimulation-evoked trials (0-20 sec; 1 stim (dark purple): 1 ± 0.22, 3 stim (medium purple): 0.46 ± 0.22, 5 stim (light purple): −0.29 ± 0.37, ordinary one-way ANOVA with Dunnett’s multiple comparisons test, *p = 0.0167, n = 4 animals). Data represented as mean ± SEM. (**j**) Normalized fluorescence minima during all stimulation-evoked trials (0-20 sec; 1 stim (dark purple): −1 ± 0.14, 3 stim (medium purple): −1.18 ± 0.2, 5 stim (light purple): −0.87 ± 0.14, ordinary one-way ANOVA with Dunnett’s multiple comparisons test, p > 0.05, n = 4 animals). Data represented as mean ± SEM. (**k**) Schematic of viral injection sites for κLight1.3a into NAcSh and ChRimson into BLA of WT or DYN-KO mice. (**l)** Representative 20X coronal image (left panel) showing expression of κLight1.3a (green), ChRimson (red), DAPI (blue), and fiber placement in NAcSh (left; scalebar – 200 μm), and ChRimson (red) and DAPI (blue) in BLA (right; scalebar – 200 μm) from 6 animals and showed similar results. (**m**) Mean recorded κLight1.3a activity averaged across all animals following i.p injections of U50,488 in WT (dark; n = 4 animals) and DYN-KO (light; n = 4 animals). Solid lines represent the mean, and shaded areas represent SEM. (**n**) Heatmap raster plot of recorded κLight1.3a activity averaged across all animals following i.p injections of U50,488 in WT (dark; n = 4 animals) and DYN-KO (light; n = 4 animals) displayed in animal ascending order by average activity following injection. (**o**) Normalized peak fluorescence during injection period (0-50 min; WT: 1 ± 0.24, DYN-KO: 0.9 ± 0.17; two tailed paired t test, p = 0.8078, n = 4 animals). Data represented as mean ± SEM. U50 = U50,488. (**p**) Normalized fluorescence area under the curve of single trails during injection period (0-50 min; WT: 1 ± 0.38, DYN-KO: 0.73 ± 0.3; two tailed paired t test, p = 0.6123, n = 4 animals). Data represented as mean ± SEM. U50 = U50,488. (**q**) Mean recorded κLight1.3a activity averaged across all trials during ChRimson stimulation-evoked trials in WT and DYN-KO (n = 4 animals). Solid lines represent the mean, and shaded areas represent SEM. Stim indicates the time of stimulus application. (**r**) Heatmap raster plot of recorded κLight1.3a activity averaged across all trials during ChRimson stimulation-evoked trials in WT and DYN-KO (n = 4 animals) displayed in ascending trial order by average activity across trials. Stim indicates the time of stimulus application. (**s**) Normalized peak fluorescence across vehicle and aticaprant treatment during all ChRimson stimulation-evoked trials (0-20 sec; WT: 1 ± 0.09, DYN-KO: 0.48 ± 0.08; two tailed paired t test, ***p = 0.0004, n = 4 animals). Data represented as mean ± SEM. (**t**) Normalized fluorescence area under the curve of single trails across vehicle and aticaprant treatment during all ChRimson stimulation-evoked trials (0-20 sec; WT: 1 ± 0.1, DYN-KO: 0.43 ± 0.05; two tailed paired t test, ****p < 0.0001, n = 4 animals). Data represented as mean ± SEM.

To detect dynorphin signaling at κOR-expressing neurons, we injected κOR-Cre mice with AAV5-CAG-DIO-κLight1.3a and implanted optical fibers in the NAcSh. A subset of mice was also injected with the red-shifted opsin ChRimson (AAV5-DIO-*EF1a*-ChRimson-tdTomato) in the BLA (Fig. 5a–c and Extended Data Fig. 5a); ChRimson-lacking mice served as a negative control to determine if optical stimulation produced artifactual dynamics in κLight1.3a fluorescence. We first examined the response of κLight1.3a to the agonist U50,488 in these mice (Fig. 5d). U50,488 (10 mg per kg body weight; i.p.) administration resulted in a rapid, sustained and robust increase in the fluorescence of κLight1.3a. This increase was significantly attenuated when the animals were pretreated with the short-acting, reversible κOR antagonist JNJ-67953964 (ref. ^51^; aticaprant, 3 mg per kg body weight; i.p.; *P* = 0.034, paired *t*-test), demonstrating the selectivity of κLight1.3a responses in vivo (normalized peak, *P* = 0.0344, paired *t*-test, normalized area under the curve (AUC), *P* = 0.0138, paired *t*-test; Fig. 5e–h).

Next, we tested whether κLight1.3a can detect endogenous dynorphin release in the NAc evoked via stimulation of glutamatergic BLA terminals, known to densely innervate the NAc^49^. A 1-s, 20-Hz, 5-ms pulse-width stimulation produced a brief artifact, followed by a significant increase in κLight1.3 fluorescence (Extended Data Fig. 5b,c). Importantly, this stimulus artifact was present to the same extent in all animals, with and without ChRimson expression in the BLA terminals (Extended Data Fig. 5d). However, the subsequent increase in κLight1.3a fluorescence was present only in the animals expressing ChRimson in BLA, suggesting that this elevation is due to the BLA terminal stimulation-evoked release of dynorphin (*P* < 0.0001, Welch’s *t*-test; Extended Data Fig. 5e). To determine the appropriate stimulation parameters for stimulation-evoked dynorphin release, we performed a battery of experiments modulating stimulation number (1–5), laser intensity (0.5–5 mW) and stimulation time (1–30 s) within the same session in a randomized order (Extended Data Fig. 5f–h). Varying the length of stimulation from 1 s to 5 s revealed, somewhat paradoxically, that 1 s of photostimulation produced the most κLight1.3a activation, while the magnitude of the artifact (fluorescence minimum) remained constant throughout (*P* = 0.0082, Brown–Forsythe and Welch ANOVA test; Extended Data Fig. 5i,j). Based on these results, we performed all our subsequent experiments using 1-s, 20-Hz, 5-ms pulse-width stimulation.

We then determined the pharmacological selectivity of BLA terminal stimulation-evoked κLight1.3a activation. We first pretreated animals with vehicle or aticaprant (3 mg per kg body weight; i.p.), followed by ten trials per animal of BLA terminal stimulation while simultaneously monitoring κLight1.3a fluorescence. We observed that κOR antagonism significantly decreased stimulation-evoked κLight1.3a activity in vivo (normalized peak, *P* = 0.0365, paired *t*-test; normalized AUC, *P* < 0.0001, paired *t*-test; Fig. 5i–l). We then posited that if this is due to κOR antagonism, wherein the antagonist prevents endogenous dynorphin from binding κLight1.3a, we should obtain a similar result following κOR agonism due to κLight1.3 occupancy by U50,488. Hence, we injected animals with vehicle or U50,488 (10 mg per kg body weight; i.p.) and performed the aforementioned recordings of stimulation-evoked κLight1.3 activity. As with aticaprant, we found that U50,488 significantly blunted evoked-κLight1.3a activation (normalized peak, *P* = 0.0022, paired *t*-test; normalized AUC, *P* = 0.0072, paired *t*-test; Fig. 5m–p). This suggests that U50,488 occupied and competed for the binding of evoked endogenous dynorphin to κLight1.3a. Next, to determine whether κLight1.3 activity was specific to dynorphin, we performed similar BLA terminal stimulation-evoked experiments in DYN-knockout (KO) mice, or WT controls (Extended Data Fig. 5k,l). As expected, injection of the exogenous receptor-specific agonist U50,488 (10 mg per kg body weight, i.p.) elicited comparable increases in κLight1.3 activity (Extended Data Fig. 5m–p). Importantly, whereas BLA stimulation-evoked κLight1.3 activity increased in WT controls, it was significantly diminished in DYN-KO animals (Extended Data Fig. 5q–t). Altogether, these results demonstrate that we can use optogenetics to trigger and measure terminal-stimulated dynorphin release with κLight in a circuit-specific manner.

### Monitoring behavior-triggered endogenous opioid release in vivo

**Fig. 6:**
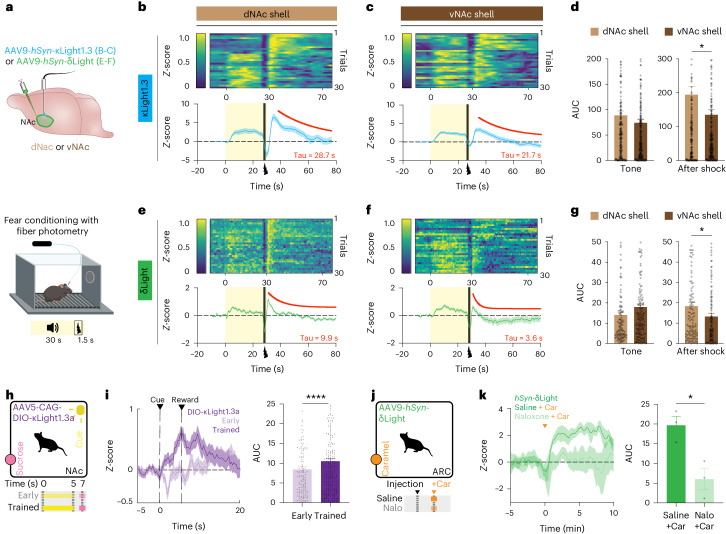
Imaging dynorphin and enkephalin dynamics during aversive and rewarding behavior. **a**, Schematics show expression of κLight1.3 or δLight in the dNAc shell or the vNAc shell (top), followed by a fear conditioning protocol during fiber photometry recording. **b**,**c**, κLight1.3 response in the dNAc (**b**) and the vNAc (**c**): Top, sorted shock trials averaged across animals from top to bottom in chronological order (trial 1 at the top, trial 30 at the bottom). Bottom, average trace of κLight1.3 response (blue) during fear conditioning, tone (0–30 s, yellow shaded area) and shock (27.5–29 s, black). Solid blue line represents the mean, and the shaded area represents the s.e.m. dNAc, *n* = 7 animals; vNAc, *n* = 8 animals. One-phase decay fit from 35 s to 80 s (red). Tau indicates the decay constant. **d**, AUC of single trials in **b** and **c** during tone and after shock. Tone AUC in dNAc: 89 ± 8.5, tone AUC in vNAc: 74 ± 7, two-tailed unpaired *t*-test, *P* = 0.1829, NS. Post-shock AUC in dNAc: 194 ± 24, post-shock AUC in vNAc: 135 ± 15, two-tailed unpaired *t*-test, *P = 0.0355. **e**,**f**, δLight response in the dNAc (**e**) and the vNAc (**f**). Experimental details same in **b** and **c**. dNAc, *n* = 4 animals; vNAc, *n* = 5 animals. One-phase decay fit from 31 s to 80 s (red). **g**, AUC of single trials in **e** and **f** during tone and after shock. Tone AUC in the dNAc: 14 ± 1.4, tone AUC in the vNAc: 18 ± 1.5, two-tailed unpaired *t*-test, *P* = 0.0582, NS. Post-shock AUC in the dNAc; 18 ± 1.8, post-shock AUC in vNAc; 13 ± 1.4, two-tailed unpaired *t*-test, *P = 0.0276. Error bars represent the s.e.m. In **d** and **g**, all single trial AUCs are plotted and compared for tone (0–25 s) and after shock (30–70 s) from *n* = 4 animals for δLight response in the dNAc and *n* = 5 animals for δLight response in the vNAc. **h**, Schematic shows classical Pavlovian conditioning. **i**, Left, mean κLight1.3a activity averaged across all trials during day 1 (early; light purple) and day 7 (trained; dark purple) of Pavlovian conditioning (*n* = 6 animals). Solid lines represent the mean, and shaded areas represent the s.e.m. Right, AUC of single trials across early and trained stages of Pavlovian conditioning; early: 8.4 ± 0.74, trained: 10.51 ± 0.77, two-tailed paired *t*-test, ****P < 0.0001. Error bars represent the s.e.m. **j**, Schematic shows caramel retrieval experiment. **k**, Left, averaged δLight activity upon caramel retrieval after injection of saline (dark green) or 4 mg per kg body weight naloxone (light green; *n* = 3 animals). Solid line represents the mean, and shaded area represent the s.e.m. Right, AUC of single trials compared between saline and naloxone conditions, saline: 20 ± 2.3, naloxone: 6 ± 2.7, *P = 0.0197, two-tailed unpaired *t*-test. Error bars represent the s.e.m.

**Extended Data Fig. 6:**
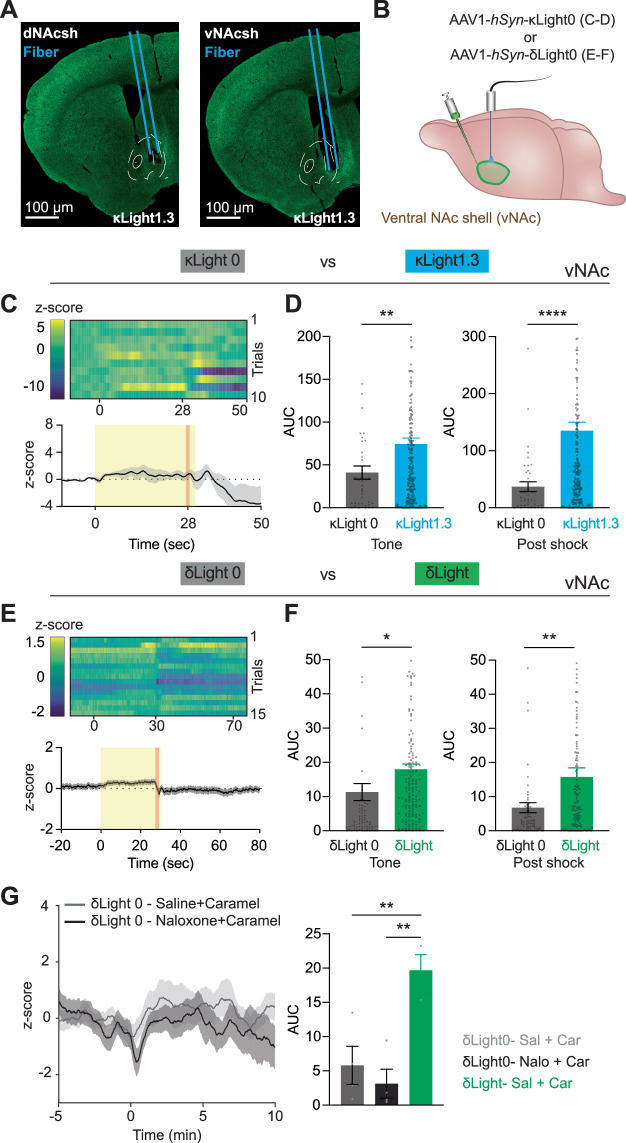
In vivo fiber photometry during aversive and rewarding behaviors with control sensors. (**a**) Representative images indicating κLight1.3 expression in dNAcsh from 7 animals (left) and vNAcsh from 8 animals (right) showing similar results. Blue lines indicate the fiber tract. Scale bar = 100 μm. (**b**) Experimental schematic indicating injection of κLight0 and δLight0 into vNAc individually, followed by fear conditioning protocol (30 sec tone, co-terminated with 1.5 sec shock, as described in Fig. 6a), recorded with fiber photometry. (**c**) κLight0 response in vNAc: (Top) Sorted shock trials averaged across animals from top to bottom in chronological order (trial 1 at the top, trial 10 at the bottom). (Bottom) Average trace of κLight0 response (black) during fear conditioning, tone (0-30 sec, yellow shade), shock, (27.5-29 sec, orange). Solid line represents the mean, and shaded areas represent SEM. n = 5 animals. (**d**) Area under the curve of single trails in (Extended Data Fig. 6c) and (Fig. 6c) during tone and post-shock. Tone AUC κLight0 (gray): 41 ± 7.6, tone AUC κLight1.3 (blue): 74 ± 7, two tailed unpaired t test, **p = 0.0016. Post-shock AUC κLight0 (gray): 37 ± 8.5, post-shock AUC κLight1.3 (blue): 135 ± 15, two tailed unpaired t test, ****p = 0.0001. κLight1.3 bar graph data reused from Fig. 6d. Error bars represent SEM. Plotted and compared all single trial AUCs for tone (0–25 sec) and post-shock (30–70 sec) from n = 5 animals for κLight0 and n = 8 animals for κLight1.3. (**e**) δLight0 response in vNAc: (Top) Sorted shock trials averaged across animals from top to down in chronological order (trial 1 at the top, trial 15 at the bottom). (Bottom) Average trace of δLight response (black) during fear conditioning, tone (0-30 sec, yellow shade), shock, (27.5–29 sec, orange). Solid line represents the mean, and shades represent SEM. n = 5 animals. (**f**) Area under the curve of single trails in (Extended Data Fig. 6e) and (Fig. 6f) during tone and post-shock. Tone AUC δLight0 (gray): 11 ± 2.5, tone AUC δLight (green): 18 ± 1.5, two tailed unpaired t test, *p = 0.025. Post-shock AUC δLight0 (gray): 6.8 ± 1.5, post-shock AUC δLight (green): 16 ± 2.7, two tailed unpaired t test, **p = 0.0034. δLight bar graph data reused from Fig. 6g. Error bars represent SEM. Plotted and compared all single trial AUCs for tone (0–25 sec) and post-shock (30–70 sec) from n = 5 animals for δLight0 and n = 5 animals for δLight. (**g**) (Left) Averaged δLight0 fluorescence upon caramel retrieval after injection of saline (gray) or 4 mg/kg naloxone (black) (n = 4 animals). Solid lines represent the mean, and shaded areas represent SEM. (Right) Area under the curve of single trails between δLight0-saline, δLight0-naloxone, and δLight-saline conditions, δLight0-saline (gray): 5.8 ± 2.8, δLight0-naloxone (black): 3.1 ± 2.1, δLight-saline (green): 20 ± 2.3, δLight-saline vs δLight0-saline **p = 0.0088, δLight-saline vs δLight0-naloxone **p = 0.0032, ordinary one-way ANOVA with Dunnett’s multiple comparisons test. δLight-saline bar graph data reused from Fig. 6k. Error bars represent SEM. Abbreviations: saline (Sal), naloxone (Nalo), caramel (Car).

After successfully detecting optogenetically evoked dynorphin release, we next sought to use κLight and δLight to monitor longitudinal opioid peptide signaling dynamics in behaving animals under fear-inducing and rewarding conditions. Previous studies have demonstrated that dynorphin neurons in ventral and dorsal NAcSh subregions (vNAc and dNAc, respectively) have a distinct role in aversive and reward behavior^47^. Furthermore, subregion-specific dynorphin and enkephalin release have been measured in vNAc versus dNAc using an opto-dialysis method^30^. We thus decided to examine the utility of κLight1.3 and δLight in probing subregion-specific release of opioid peptides in the NAc during fear learning. To do so, AAV9-*hSyn*-κLight1.3 or AAV9-*hSyn*-δLight was injected in the dNAc and the vNAc, followed by fiber implantation. Three weeks after surgery, we measured peptide transients during an auditory fear conditioning experiment consisting of 30 presentations of a 30-s tone co-terminating with a 1.5-s foot shock (0.5 mA; Fig. 6a and Extended Data Fig. 6a). In the case of κLight, both dNAc and vNAc, we observed a quick rise in fluorescence intensity after the onset of the tone, which was sustained during tone presentation, followed by a small dip at the onset of the shock and a large rise immediately after the foot shock. The fluorescence signal then gradually decreased to the baseline after ~40 s (Tau - κLight1.3 in dNAc = 28.7 s; Tau - κLight1.3 in vNAc = 21.7 s; Fig. 6b,c). To assess differences in release between NAc subregions, we calculated the AUC of individual trials. The AUC to the tone was similar between dNAc and vNAc, whereas the AUC of the post-shock response was significantly higher in the dNAc compared to the vNAc (AUC dNAc, 194 ± 24; AUC vNAc, 135 ± 15, *P* = 0.0355, unpaired *t*-test; Fig. 6d). We did not observe fluorescence changes during fear learning when AAV1-*hSyn*-κLight0 was expressed either in the dNAc or the vNAc (Extended Data Fig. 6b–d).

In the case of δLight in the dNAc, we observed a brief increase in fluorescence triggered by the tone that gradually decreased to the baseline during tone presentation. The foot shock also triggered a large fluorescence increase followed by a sharp decay over 10 s after the shock (Tau - δLight in dNAc = 9.9 s; Tau - δLight in vNAc = 3.6 s; Fig. 6e,f). Although the AUC of the tone-evoked response in the vNAc was slightly larger in amplitude than in the dNAc, the difference was not significant. Again, the AUC of the shock-evoked response in the dNAc was significantly higher than in the vNAc (AUC dNAc; 18 ± 1.8, AUC vNAc; 13 ± 1.4, *P* = 0.0276, unpaired *t*-test; Fig. 6g). We observed significantly attenuated fluorescence changes to the tone and shock in the animals expressing the control sensor δLight0 (Extended Data Fig. 6e,f).

Together, these data suggest κLight and δLight can faithfully report the subregional differences in endogenous opioid peptide release triggered during fear learning. More interestingly, the post-shock signals from κLight were much larger and longer lasting in early trials, and the response gradually shifted from the shock to tone as the number of trials increased (Fig. 6b,c). We did not observe this pattern of signal shifting from shock to tone in δLight (Fig. 6e,f), which suggests that opioid peptide, such as dynorphin, might actively track fear state in the NAcSh.

To determine the utility of κLight to probe reward-trigger endogenous dynorphin release, we first recorded the response of κLight1.3a to Pavlovian conditioning in the NAc (Fig. 6h). To target κOR-expressing neurons, we again injected CAG-DIO-κLight1.3a into the NAc of κOR-Cre mice and trained these animals using classical reward conditioning. Although reward delivery during early trials did not produce fluorescence increases, we found a significant increase in κLight1.3a fluorescence during reward delivery and consumption following conditioning, as animals increased their reward consumption across training (AUC early: 8.4 ± 0.739, AUC trained: 10.51 ± 0.77, *P* < 0.0001, paired *t*-test; Fig. 6i). These results suggest that endogenous dynorphin is released during reward reinforcement, supporting our prior work showing that subpopulations of dynorphin neurons in the NAcSh are reinforcing^47^.

Similarly, we monitored δLight fluorescence in the ARC while mice retrieved caramel rewards (Fig. 6j). We observed elevated δLight signals in animals injected with saline following caramel retrieval, and this response was blocked when naloxone (4 mg per kg body weight) was injected before caramel retrieval (AUC saline: 20 ± 2.3, AUC naloxone: 6 ± 2.7, *P* = 0.0197, unpaired *t*-test; Fig. 6k). We did not observe an increase in δLight0 in response to caramel retrieval under either condition (Extended Data Fig. 6g). Together, these results suggest that κLight and δLight can faithfully track dynamic changes in endogenous opioid release during the full course of aversive and rewarding behaviors in vivo.

## Discussion

In this study, we develop and characterize genetically encoded opioid receptor sensors for high-resolution tracking of opioid peptides under various experimental settings. G-protein-coupled-receptor-based sensors have been valuable in monitoring neuromodulator signals in awake animals^52,53^, initially for biogenic amines and acetylcholine^35,54–60^, and more recently for NPs including oxytocin, orexin and others^42,61–63^. The development of opioid sensors addresses a crucial need in the neuroscience toolkit due to opioids’ widespread significance.

All three sensors, µLight, κLight and δLight, collectively respond to a wide range of opioid ligands, including endogenous opioid NPs, with κLight and δLight retaining the pharmacological selectivity of the parent receptor. These sensors can detect and differentiate conformational changes of the receptor induced by various peptides, which is difficult to do using traditional radioligand binding assays. The systematic characterization of pharmacological profiles provided by these sensors could open new doors for imaging-based high-throughput screening of a chemical library targeting opioid signaling. However, µLight is weakly activated by small-molecule drugs like morphine and fentanyl and has a lower binding efficacy for endogenous peptides. In fact, oxycodone was observed to suppress µLight fluorescence, which could indicate oxycodone activates µOR with a different conformation. This could lead to the application of performing drug screening to select for compounds that activate µOR in a desired conformation. Structural studies of µORs in active and inactive states revealed that conformational changes of TM5 and TM6 depend on an allosteric coupling between ligand-binding pockets and G-proteins^64^. As cpGFP was inserted into ICL3, it is possible that cpGFP insertion decreased such coupling. Future optimization of µLight is crucial for reliably detecting µOR-selective NP β-endorphin.

NP receptors can be expressed at a considerable distance (µm–mm) from putative peptide release sites, suggesting volume transmission as one mode of neuropeptidergic transmission, enabling small amounts of NPs to widely impact brain function. We used κLight with spatially restricted peptide photorelease to measure DynA8 in the dStr, indicating that it can signal via volume transmission to activate receptors over 100 µm away within seconds, with an apparent diffusion coefficient of 1.4 µm^2^ s^−1^. Diffusion coefficients for NPs and similar molecules vary greatly depending on peptide type and brain region, peptidase content, as well as tissue tortuosity^8,62,65–67^. We measured diffusion in the striatum, a tortuous region with myelinated fiber bundles and patch-matrix microcircuits; peptides may exhibit higher mobility in less tortuous regions. Although peptide uncaging has advantages, it doesn’t target endogenous release sites and may release larger quantities than dense-core vesicles. Additionally, confining sensor expression to κOR-expressing cells could improve sensitivity to endogenous peptide release by minimizing background fluorescence from neurons potentially unexposed to locally released peptide, which can further enhance the accuracy of measurement. Further studies on endogenously released peptide spread are needed.

Understanding the neural activity patterns required for evoking NP release remains a decades-long challenge. Monitoring NP release in response to electrical or optogenetic stimulation ex vivo or in vivo offers a powerful method for identifying these activity patterns. We demonstrated κLight’s utility in determining electrical parameters to trigger endogenous release in hippocampal slices; this overcomes the challenge of using electrophysiological assays for endogenous receptor activation.

Identifying conditions that support endogenous peptide release may be most appropriately addressed in vivo, where neural circuits remain fully intact and endogenous neuromodulatory tone remains unaltered by brain slicing. To demonstrate circuit and cell-type-specific release, rather than stimulating dynorphinergic cells within the NAc directly, we optogenetically stimulated their glutamatergic inputs arising from the BLA. Prior work has established that optogenetic stimulation of BLA terminals in the NAc reliably drives action potentials in striatal neurons in brain slices, as well as facilitate reward-seeking behavior^49^. In addition, synaptic stimulation of action potentials in peptidergic neurons via strong glutamatergic drive can activate metabotropic glutamate receptors, which have been implicated as gatekeepers for dynorphin secretion from striatal neurons in brain slices^68^. Using this optogenetic approach, we successfully identified stimulation conditions that result in κLight activation, presumably via dynorphin secretion from striatal neurons, as BLA neurons themselves express little to no prodynorphin mRNA (Allen Brain Atlas ISH data). Paradoxically, we found that increasing the duration of the stimulus beyond 1 s decreased the degree of κLight activation. This is likely due to the stimulation artifact generated by red light, as evidenced by the comparable minima observed in the animals expressing ChRimson and the controls that lack it (Extended Data Fig. 5b–d). Furthermore, increasing stimulation number also increased the width of the artifact (Extended Data Fig. 5f–h). While our data strongly suggest that this paradoxical suppression is likely due to stimulation artifacts induced by red light, future studies are required to explore the possibility of recruitment of additional neurochemical signaling processes during sustained stimulation that may suppress dynorphin release. Moreover, κOR-mediated suppression of BLA synaptic output, via dynorphin–κOR signaling at BLA terminals following sustained stimulation, resulting in the dampening of further synaptic activation of NAc dynorphin neurons requires further study. Additionally, whether prolonged stimulation of opioid release, either via optogenetics, or in response to strong behavioral stimuli such as foot shock, results in the transient quenching of sensor activity also warrants future exploration.

In this study, we further demonstrated the utility of κLight and δLight sensors in tracking rapid dynamic changes in endogenous opioid peptide release triggered by both reward and aversion, which can vary between subregions. Collectively, κLight and δLight respond to most endogenous opioid NPs, including various dynorphin forms and enkephalins. However, the promiscuity among opioid receptors and peptides presents a disadvantage in specificity, as the sensors cannot reliably distinguish between the endogenous peptides that might activate them—indeed many brain areas are rich in multiple opioid NPs. While our data using DYN-KO animals suggest a good degree of specificity for κLight to detect endogenous dynorphin (Extended Data Fig. 5m–t), further engineering efforts combined with structural analysis may make it possible to reduce such promiscuity. We expect broad application of these opioid sensors to enable a new understanding of how endogenous opioid peptide signaling contributes to various physiological and pathological conditions, including pain, stress, reward and drug addiction. Monitoring circuit-specific peptide release in behaving animals may reveal new opioid functions in behavioral state transitions and associative learning. Detecting discrete peptide release events, evoked optogenetically or behaviorally, can help identify differences in opioid secretion under various pharmacological, behavioral or disease states. While pharmacology, photopharmacology and optogenetics have contributed substantially to opioid receptor signaling knowledge, these sensors enable a shift from focusing on receptor activation consequences to exploring endogenous NP secretion’s impact on the brain’s complex and diverse functions.

## Methods

### Animals

Animals were housed in ventilated home cages with at most five mice per cage. Animals were housed in a vivarium with a 12-h light and 12-h dark standard light cycle, with a temperature of 68–79 °F and humidity of 30–70%. All housing and experimental procedures involving animals were approved by the Institutional Animal Care and Use Committee at the University of California, Davis, the University of California San Diego, the University of Washington, the University of Iowa Cold Spring Harbor Laboratory and the National Institute of Mental Health or Icahn School of Medicine and adhered to principles described in the National Institutes of Health (NIH) Guide for the Care and Use of Laboratory Animals. The University of California Davis, the University of California San Diego, the University of Washington, the University of Iowa, Cold Spring Harbor Laboratory, the National Institute of Mental Health and Icahn School of Medicine are accredited by the Association for Assessment and Accreditation of Laboratory Animal Care International (AAALAC).

### Reagent and key resources

All reagent, software and equipment are summarized in Supplementary Table 1.

### Sensor development and characterization

#### Development of κLight, δLight and µLight

All constructs were designed using circular polymerase extension cloning, restriction cloning and gBlock gene fragments (Integrated DNA Technologies)^69^. Sequences coding for a FLAG epitope were placed at the 5′-end of the construct as previously described^70^. *HindIII* and *NotI* cut sites were placed at the 5′- and 3′ ends, respectively, for cloning into pCMV (Addgene) to generate all pCMV constructs. *BamHI* and *HindIII* sites were introduced via PCR for final subcloning onto pAAV.hSynapsin1 vectors (Addgene). To maximize coupling between conformational changes and chromophore fluorescence, we chose to use a cpGFP module (LSS-LE-cpGFP-LP-DQL) from GCaMP6 (ref. ^71^) for insertion into the human OPRK1 (κOR), OPRD1 (δOR) and OPRM1 (µOR) using circular polymerase extension cloning.

For screening linker variants, we generated linker libraries by first creating an insert DNA carrying a randomized two-amino-acid linker on each side of cpGFP (LSS-xx-cpGFP-xx-DQL). Cloned constructs were amplified and purified with the Qiagen PCR purification kit before NEB 5-α competent *Escherichia coli* transformation. Competent cells were plated onto kanamycin-containing agar plates. After allowing for 24 h of growth at 37 °C, single colonies were manually picked and grown overnight as described previously^72^. Plasmids from the colonies were purified using the Qiagen miniprep kit. Top variants were sequenced by Genewiz. For the iteration of κLight variants, κLight1.1 was discovered after linker screen and resulted in linker GI-PH. κLight1.2a: V164K from κLight1.1. κLight1.2b is κLight1.2a with ER2 tag. κLight1.2c is κLight1.2b with T603K. κLight1.3 is κLight1.2a with TlcnC, PRC and ER2 tag. κLight0 is κLight1.2a with D128N. δLight has linker GI-PH, p.Val154Lys mutation with PRC and ER2 tag. δLight0 has p.Asp128Asn mutation. µLight has linker sequence CI-SH, p.Val175Gln mutation with PRC and ER2 tags. To make AAV plasmids, NEB stable competent cells were transformed with pAAV plasmids. After growth on an agar plate at 30 °C, a single colony was selected. After sequencing confirmed the presence of the sensor gene, the cells were expanded at 30 °C in 100 ml of growth medium (2xYT) and purified with a Qiagen Endo-free Plasmid Maxi Kit and sent to the UC Davis Virus Packaging Core for virus production.

#### Tissue culture

HEK293T cells were grown in DMEM, supplemented with FBS and penicillin–streptomycin. Cells were transfected with Effectene according to the manufacturer’s instructions. Before imaging, cells were washed with Hank’s Balanced Salt Solution (HBSS) supplemented with 2 mM MgCl_2_ and 2 mM CaCl_2_. All images were collected in HBSS containing Mg^2+^ and Ca^2+^ (HBSS+).

#### Transient transfection

HEK293T cells were plated and transfected concurrently 24 h before each experiment using the Qiagen Effectene Transfection Reagent kit according to the manufacturer’s protocol.

#### Displacement binding assays

Membranes were prepared from µLight, δLight, κLight cells or CHO-µOR cells as described previously^37^. Displacement binding assays were carried out with membranes (100 µg), [^3^H]diprenorphine (3 nM final concentration) and different ligands (0–10 µM final concentration) as described previously^16,38^, except that the assay buffer consisted of 50 mM Tris-Cl (pH 7.4) containing 100 mM NaCl, 10 mM MgCl_2_, 0.2 mM EGTA and protease inhibitor cocktail (Sigma-Aldrich, P2714), and incubation was carried out for 1 h at 30 °C.

#### Micro-confocal high-throughput imaging experiments

Glass-bottom 96-well plates (P96-1.5H-N, Cellvis) were coated with 50 µg ml^−1^ of poly-d-lysine (Sigma, P6407-5MG) and 10 µg ml^−1^ of laminin (Sigma, L2020) overnight in an incubator (37 °C, 5% CO_2_). Plates were washed with Dulbecco’s PBS (Thermo Fisher, 14190-250) and PSYLI2 cells were suspended in DMEM (Fisher, 11995073) containing 10% FBS (Fisher, 26-140-079) with 5% penicillin–streptomycin (Fisher, 15140-163) and plated at a density of 40,000 cells per well 24 h before each experiment. Immediately before an experiment, stock solutions of drugs in dimethylsulfoxide (10 mM) were diluted at a 1:100 ratio in imaging media distributed across an empty 96-well plate (treatment plate) in triplicate following a randomized plate map. The imaging media consisted of 1× HBSS (Fisher, 14175103) containing 0.5 M MgCl_2_ (Sigma, M8266-1KG) and 0.5 M CaCl_2_ (Sigma, C5670-50G). Cells grown in a separate 96-well plate (assay plate) were gently washed 3× with imaging media, and the wells were filled with an appropriate volume of imaging media for the respective experiment (see below).

#### HEK cell titration

For titration experiments, 50 µl of imaging medium was added to each well of the assay plate. Wells were then imaged with ImageXpress Micro Confocal High-Content Imaging System at ×40 (N.A., 0.6) with four regions of interest (ROIs) taken per well with no bias to location and no overlap of the ROIs (exposure, 300 ms) with MetaXpress software. Next, 50 µl from the treatment plate was transferred to the assay plate containing a double desired final concentrations. As for titration dose and controls, ligand of interest from 1 pM to 100 µM (final) dissolved in HBSS+ as vehicle were used. Blank controls with vehicles were present on every plate with randomized locations. After 5 min of incubation, the same sites were reimaged using the same settings.

Once imaging was complete, the images were exported and analyzed using a self-written MATLAB script. The script will be deposited in GitHub. In short, segmentation was performed on individual images and a mask highlighting the membrane of the HEK293T cells was generated. Pixel intensities were obtained from the mask-highlighted area and exported into Excel. The Δ*F/F* values for each well were calculated using the following equation:

$${\frac{(\mathrm{average}\; \mathrm{fluorescence}\; \mathrm{after}\; \mathrm{drug}-{\mathrm{average}\; \mathrm{fluorescence}\; \mathrm{before}\; \mathrm{drug}})}{\mathrm{average}\; \mathrm{fluorescence}\; \mathrm{before}\; \mathrm{drug}}}$$

These values were then used to obtain the triplicate mean (*n* = 3).

SNR values are calculated by:

$${\frac{(\mathrm{average}\; \mathrm{fluorescence}\; \mathrm{after}\; \mathrm{drug}-\mathrm{average}\; \mathrm{fluorescence}\; \mathrm{before}\; \mathrm{drug})}{\surd \mathrm{average}\; \mathrm{fluorescence}\; \mathrm{before}\; \mathrm{drug}}}$$

#### Schild regression analysis

A treatment plate was prepared by premixing various concentrations of antagonists with increasing concentrations of the sensors’ specific agonist. The agonist and antagonist were premixed in doubled concentrations in a treatment plate in HBSS+. Wells were first imaged with 50 μl HBSS+ in the well, and 50 µl of the mix of ligands from the treatment plate was then added to the imaging plate, and the wells were imaged again under the same settings.

#### cAMP assay

HEK293 cells (American Type Culture Collection), maintained in DMEM (Invitrogen, 11965118) supplemented with 10% FBS (Clontech), were transfected in 10-cm dishes with 1 μg of Flag- κLight 1.3, δLight, Flag-δOR or Flag-κOR construct cloned into pcDNA3.0, together with 2 μg of pGloSensor-20F plasmid (Promega), using Lipofectamine 2000 (Thermo Fisher, 11668019) per the manufacturer’s instructions. Twenty-four hours after transfection, cells were lifted using TrypLE Express (Thermo Fisher, 12604021), pelleted (500*g* for 5 min) and resuspended at 100,000 cells per ml in phenol red-free DMEM (Invitrogen, 31053028) supplemented with 30 mM HEPES and 250 μg per ml luciferin (Biogold). Then, 150 μl of the suspension was added to each well of a 96-well dish, and cells were maintained for 45–60 min in a 37 °C incubator. Cells were then assayed using a scanning plate-reading luminometer (Tecan Spark) at 37 °C. Baseline luminescence was measured over 5 min. Forskolin and dynorphin A (1-17; Anaspec, A24298) were used to assess κLight1.3 relative to κOR, and forskolin and DADLE (DADLE [D-Ala2, D-Leu5]-enkephalin; Sigma-Aldrich, E7131) were used assess δLight relative to δOR. Forskolin and each opioid agonist peptide were added together to each well in a volume of 50 μl, adjusted to achieve a final concentration of 10 μM forskolin and the indicated concentration of opioid agonist. Luminescence measurement was resumed for 10 min and a maximum baseline-subtracted value was used to determine the cellular cAMP response. Each condition was measured in triplicate wells and values reported were averages from four to five independent experiments.

#### β-Arrestin recruitment assay

HEK293 cells were transfected in 6-well dishes with 200 ng of Flag-κLight1.3, Flag-δLight, Flag-κOR or Flag-δOR constructs cloned into pcDNA3.0, 100 ng LargeBit-CAAX and 500 ng SmallBit β-Arrestin1 (ref. ^73^), using Lipofectamine 2000 per the manufacturer’s instructions. Twenty-four hours after transfection, cells were lifted, washed by centrifugation as above and resuspended to a density of 0.5–1 × 10^6^ cells per ml in phenol red-free DMEM supplemented with 30 mM HEPES and 5 μM coelenterazine-H (Research Products International). Cells were distributed in a 96-well dish at 150 µl per well, incubated for 15 min at 37 °C to load coelenterazine-H and then a 5-min baseline was measured in the luminometer at 37 °C. Following this, 1 µM of dynorphin A (1-17; for κLight/KOR assessment) or DADLE (for δLight/δOR assessment) was added, and measurement was resumed for an additional 30 min. The maximum baseline-subtracted luminescence was used to determine recruitment values. Each condition was measured in triplicate wells and values reported are averages from three independent experiments.

### Slice experiments

#### Stereotaxic intracranial injection

Male and female C57/B6J mice (postnatal day 0–3) were anesthetized with isoflurane and placed in a small animal stereotaxic frame (David Kopf Instruments). After puncturing the skin and skull under aseptic conditions, AAVs were injected (0.5–1 µl total volume) bilaterally through a pulled glass pipette at a rate of 100 nl min^−1^ using a UMP3 microsyringe pump (World Precision Instruments). Depending on the size of the mouse, injection coordinates ranged from 0 mm to +0.5 mm from bregma, 0.5 mm to 1.0 mm lateral and 1.8 mm to 2.3 mm below pia for dStr. For targeting hippocampus to study buffering, injection coordinates were +0.3 mm to 0.5 mm from lambda, 2.2 mm to 2.5 mm lateral and 1.4 mm to 2.0 mm below pia. After surgical procedures, mice were returned to their home cage for >30 days to allow for maximal gene expression. For CA3 injection in hippocampus for electrical stimulation, we used the coordinates: −1.7 mm AP, 1.75 mm ML, −2.3 mm DV. To achieve sparse labeling of neurons in CA3, we injected AAV1-CAG-DIO-κLight1.3a into CA3 with a 1:1,000 dilution of AAV1-*hSyn*-Cre virus. Male and female C57/B6J mice were injected 8–10 weeks postnatally.

#### Brain slice preparation

P30–60 mice were anesthetized with isoflurane and killed, and the brains were removed, blocked and mounted in a VT1000S vibratome (Leica Instruments). For striatal imaging experiments, coronal slices (300 μm) were prepared in 34 °C artificial cerebrospinal fluid (aCSF) containing 127 mM NaCl, 2.5 mM KCl, 25 mM NaHCO_3_, 1.25 mM NaH_2_PO_4_, 2 mM CaCl_2_, 1 mM MgCl_2_ and 25 mM glucose, with osmolarity of 307, equilibrated with 95% O_2_/5% CO_2_. For hippocampal electrophysiology recordings, horizontal slices (300 μm) were prepared in ice-cold choline-aCSF containing 25 mM NaHCO_3_, 1.25 mM NaH_2_PO_4_, 2.5 mM KCl, 7 mM MgCl_2_, 25 mM glucose, 1 mM CaCl_2_, 110 mM choline chloride, 11.6 mM ascorbic acid and 3.1 mM pyruvic acid, equilibrated with 95% O_2_/5% CO_2_. Slices were transferred to a holding chamber with oxygenated aCSF and incubated at 32 °C for 30 min and then left at room temperature until recordings were performed.

#### Fluorescence imaging with peptide uncaging

All video recordings were performed within 5 h of slicing in a submerged slice chamber perfused with aCSF warmed to 32 °C and equilibrated with 95% O_2_/5% CO_2_. Sensor-expressing tissue in the dStr was located and imaged through an eGFP filter cube (Semrock, GFP-3035D-OMF) under a ×60, 0.8-NA objective using a SciCam CCD camera (Scientifica) and illumination with the 470-nm LED (CoolLED). Ocular image acquisition software (QImaging) was used to acquire videos using a 100-ms exposure time at a frame rate of 1 Hz. For uncaging trials, 5 µM of CYD8 was circulated in the bath before beginning video acquisition. During uncaging trials, ScanImage was used to trigger video acquisition and the UV laser. Uncaging was carried out using 50-ms flashes of light from a 355-nm laser (DPSS Lasers). For full-field uncaging (Fig. 3c,d and Extended Data Fig. 3b,c), a 70-µm-diameter area of tissue was illuminated with collimated UV light at a power density of 5 µW/µm^2^, as measured in the sample plane. When measuring DynA8 diffusion, a 25-µm-diameter area of focused 355-nm light at a power density of 39 µW/µm^2^ was applied near the edge of the imaging field.

#### Electrophysiology

All recordings were performed within 5 h of slicing in a submerged slice chamber perfused with aCSF warmed to 32 °C and equilibrated with 95% O_2_/5% CO_2_. Whole-cell voltage-clamp recordings were obtained with an Axopatch 700B amplifier (Molecular Devices). Data were sampled at 10 kHz, filtered at 3 kHz and acquired using National Instruments acquisition boards and a custom version of ScanImage written in MATLAB (Math Works). Cells were rejected if holding currents exceeded −200 pA or if the series resistance (<25 MΩ) changed during the experiment by more than 20%. For recordings measuring inhibitory synaptic transmission in mouse hippocampus, patch pipettes (2.8–3.5 MΩ) were filled with an internal solution containing 135 mM CsMeSO_3_, 10 mM HEPES, 1 mM EGTA, 3.3 mM QX-314 (Cl − salt), 4 mM Mg-ATP, 0.3 mM Na-GTP and 8 mM Na_2_ phosphocreatine (pH 7.3, 295 mOsm kg^–^^1^). Cells were held at 0 mV to produce outward currents. Excitatory transmission was blocked by the addition to the aCSF of NBQX (2,3-dioxo-6-nitro-7-sulfamoyl-benzo[f]quinoxaline, 10 μM) and CPP (3-(2-carboxypiperazin-4-yl)propyl-1-phosphonic acid, 10 μM). To electrically evoke IPSCs, stimulating electrodes pulled from theta glass with ~5-μm tip diameters were placed at the border between stratum pyramidale and stratum oriens nearby the recorded cell (~50–150 μm), and two brief pulses (0.5 ms, 50–300 μA, 50-ms interval) were delivered every 20 s. Uncaging was carried out using 5-ms flashes of collimated full-field illumination with a 355-nm laser at different power densities, which were measured at the sample plane.

#### Data analysis

Video acquisition data were first analyzed in ImageJ and subsequently plotted in Igor Pro (Wave Metrics). The mean brightness of each frame was divided by the average baseline fluorescence of the first minute to calculate Δ*F/F*. Then, the first minute before uncaging was fit with a bi-exponential curve to estimate the rate of bleaching during the video acquisition. The fitted bleaching curve was then subtracted from the recorded traces to correct for bleaching. A 3,800 µm^2^ circle ROI was drawn at the center of the uncaging field and the mean brightness of this ROI was plotted per frame. Electrophysiology data were analyzed in Igor Pro (Wave Metrics). Peak current amplitudes were calculated by averaging over a 2-ms window around the peak IPSC. To determine the magnitude of modulation by DynA8 photorelease (the percentage of IPSC suppression), the IPSC peak amplitude measured immediately after a flash was divided by the average peak amplitude of the three IPSCs preceding the light flash. To determine the time constant of recovery (tau off), the IPSC amplitudes were fit to a monoexponential function starting at the point of maximal IPSC suppression to the point at which the IPSC amplitude returned to baseline.

#### Diffusion coefficient calculation

Based on a derivation of Fick’s law of diffusion that yields ${\gamma }_{i}^{2}=4{D}^{* }({t}_{i}+{t}_{0})$ (ref. ^66^), D* is the slope of the linear regression between *γ*^2^/4, where gamma is the half-width of the spatial fluorescence profile, and time (*t*), as demonstrated by diffusion of dextrans molecules or quantum dots in the cortex^74^. To reduce noise, we averaged 50 pixels in the *y* axis around the center line of the image plane (parallel to the uncaging spot).

#### Brain slices for two-photon imaging

Three to four weeks after viral injection, samples from adult mice were anesthetized with 2.5% avertin and perfused in ice-cold carborgen (95% O_2_ and 5% CO_2_) gassed cutting NMDG-HEPES aCSF solution that contained: 92 mM NMDG, 2.5 mM KCl, 1.25 mM NaH_2_PO_4_, 30 mM NaHCO_3_, 20 mM HEPES, 24 mM d-glucose, 2 mM thiourea, 5 mM sodium ascorbate, 3 mM sodium pyruvate, pH adjusted to 7.3–7.4 mM and supplemented with 0.5 mM CaCl_2_ and 10 mM MgCl_2_, before decapitation. Brains were quickly extracted and were cut (300 μm) with a vibratome (V1200, Leica) in ice-cold oxygenated NMDG-HEPES aCSF. Brain slices were incubated at 34–36 °C for 10 min before transferring to HEPES holding aCSF that contained 92 mM NaCl, 2.5 mM KCl, 1.25 mM NaH_2_PO_4_, 30 mM NaHCO_3_, 20 mM HEPES, 25 mM d-glucose, 2 mM thiourea, 5 mM sodium ascorbate and 3 mM sodium pyruvate, pH adjusted to 7.3–7.4 and supplemented with and 2 mM CaCl_2_ and 2 mM MgCl_2_, saturated with 95% O_2_ and 5% CO_2_ (ref. ^75^). Imaging was carried out at room temperature using a two-photon microscope. The sensor was excited at 920 nm with a titanium–sapphire laser (Ultra II, Coherent) that was focused by an Olympus ×40, 0.8-NA water immersion objective. Emitted fluorescence was separated by a 525/50-nm filter set, and detected by a photomultiplier (H7422PA-40, Hamamatsu). Data were acquired and collected with ScanImage5 software. Electrical stimulation was performed with a bipolar stimulating electrode (Array of 2 SNEX-100 PI concentric electrodes epoxied side-by-side, MicroProbes). The area within approximately 20 μm of the electrode was imaged. Rectangular voltage pulses were applied through a nine-channel programmable pulse stimulator (Master-9, A.M.P. Instruments) and a stimulus isolation unit (Analog Stimulus Isolator, A-M Systems). Imaging and electrical stimulation were controlled by an Axon Digidata 1550B. Field potentials were applied at 1, 5, 10 and 15 trains with an interstimulus interval of 0.5 s, where one single stimulus is one train at 5 V, 50 Hz with a duration of 1 s. Experiments were carried out at a scan rate of 30 Hz (512 × 512 pixels). Drugs were dissolved as a stock solution in imaging HBSS buffer and diluted to final concentration before application in the perfusion system.

Fluorescence intensities from video acquisition for two-photon field stimulation recording were performed with ImageJ. Due to the long-range diffusion nature of NPs, whole two-photon field-of-view fluorescence intensity was extracted instead of applying segmentation for fine cellular components. ∆*F/F* were calculated by (fluorescence intensity per frame − average base line fluorescence intensity) / average base line fluorescent intensity), where baseline is defined as first 2 min for each recording. The peak intensities were determined by averaging the ∆*F/F* values from frames at the plateau of elevated signals following stimulation.

The *z*-score image was processed by ∆*F*/s.d. (baseline); baseline is the average intensity of the first 500 frames without stimulation, and ∆*F* is calculated as the difference between averaged fluorescence intensity across frames at responses plateau and baseline. Data were averaged per stimulation condition and plotted by Prism.

### In vivo recordings

#### Experimental subjects and stereotaxic surgery

Adult (25–35 g), 12- to 16-week-old κOR-Cre mice or C57/B6J mice were group housed, given access to food pellets and water ad libitum and maintained on a 12-h:12-h light–dark cycle (lights on at 7:00). All animals were kept in a sound-attenuated, isolated holding facility in the lab 1 week before surgery, after surgery and during the behavioral assays to minimize stress.

For surgery, mice were anesthetized in an induction chamber (2–4% isoflurane) and placed into a stereotaxic frame (Kopf Instruments, 1900) where they were maintained at 1–2% isoflurane. Male and female mice were anesthetized, following which we performed a craniotomy and unilaterally injected as described below, using a blunt neural syringe (65457-01, Hamilton Company). For photostimulation experiments and κLight Pavlovian conditioning experiments: 300–400 nl of AAV5-DIO-ChrimsonR-tdTomato (UW NAPE Center Viral Vector Core, viral titer 5 × 10^12^ viral genomes (vg) per ml) into the BLA (stereotaxic coordinates from bregma: −1.3 mm [AP], ±3.2 mm [ML], −4.6 mm [DV]), and AAV-DIO-κLight1.3 (UC Davis Viral Core, viral titer 3.6 × 10^13^ vg per ml) followed immediately by fiber-optic implantation into the NAcSh (stereotaxic coordinates from bregma: +1.3 mm [AP], ±0.5 mm [ML], −4.5 mm [DV]). For experiments involving the DYN-KO, AAV5-DIO-ChrimsonR-tdTomato and AAV-DIO-κLight1.3 were mixed with AAV-DIO-Cre at a 1:1 dilution and injected and implanted in the aforementioned regions using the same coordinates. For fear conditioning experiments: 300–400 nl of AAV9-*hSyn*-κLight1.3 (Canadian Neurophotonics, viral titer 1 × 10^13^ vg per ml) and AAV9-*hSyn*-δLight (Canadian Neurophotonics, viral titer 3.3 × 10^12^ vg per ml) were injected separately into the dorsal NAcsh (dNAcsh, +1.3 mm [AP], ±0.5 mm [ML], −4.5 mm [DV]) and ventral NAcsh (vNAcsh, +1.3 mm [AP], ±0.5 mm [ML], −5 mm [DV]). For fear conditioning control experiments: 300–400 nl of AAV1-*hSyn*-κLight0 (Canadian Neurophotonics, viral titer 7.8 × 10^12^ vg per ml) and AAV1-*hSyn*-δLight0 (Canadian Neurophotonics, viral titer 9.5 × 10^12^ vg per ml) were injected into vNAcsh as controls. For δLight caramel reward retrieval experiments: 300–500 nl virus (AAV9-*hSyn*-δLight, AAV-syn-δLight0) was injected bilaterally in the mediobasal hypothalamus (ARC, −1.25 mm [AP], ±0.25 mm [ML], −5.6 mm [DV] from the surface of the brain) using a pulled glass pipette (Drummond Scientific, Wiretrol) controlled by a micromanipulator (Narishige). A fiber cannula was then implanted at the injection site, and the implants were secured using two bone screws and a dental cement head cap (Lang Dental). ([AP] values were measured from bregma, and [ML] values were measured from the skull at bregma unless otherwise noted.)

#### Fiber photometry

For fiber photometry studies, recordings were obtained throughout the entirety of drug injection, Pavlovian conditioning and head-fixed sessions as previously described^76^. Before recording, an optic fiber was attached to the implanted fiber using a ferrule sleeve (Doric, ZR_2.5). Two LEDs were used to excite κLight1.3. A 531-Hz sinusoidal LED light (Thorlabs, LED light: M470F3; LED driver: DC4104) was bandpass filtered (470 ± 20 nm, Doric, FMC4) to excite κLight1.3 and evoke emission. A 211-Hz sinusoidal LED light (Thorlabs, LED light: M405FP1; LED driver: DC4104) was bandpass filtered (405 ± 10 nm, Doric, FMC4) to evoke isosbestic control emission. Laser intensity for the 470-nm- and 405-nm-wavelength bands were measured at the tip of the optic fiber and adjusted to 50 μW before each day of recording. κLight1.3 fluorescence traveled through the same optic fiber before being bandpass filtered (525 ± 25 nm, Doric, FMC4), transduced by a femtowatt silicon photoreceiver (Newport, 2151) and recorded by a real-time processor (TDT, RZ5P). The envelopes of the 531-Hz and 211-Hz signals were extracted in real time by the TDT program Synapse at a sampling rate of 1,017.25 Hz. For the ChrimsonR stimulation experiments, a 625-nm laser was used at 2 mW of intensity to deliver red light through the tip of the same optic fiber used to excite BLA terminals for stimulation-evoked dynorphin release.

#### Drug injection

Mice were pretreated with either vehicle (17:1:1:1 ratio of saline:dimethylsulfoxide:corn oil:ethanol) or aticaprant (Eli Lilly) at 3 mg per kg body weight of body weight i.p. for 30 min. Mice were then tethered to a photometry cable and placed in a chamber. Following a 5-min baseline recording, mice were injected with either saline or U50,488 (Sigma-Aldrich) at 10 mg per kg body weight body weight i.p. Recordings were conducted for a total of 1 h.

#### Pavlovian behavior paradigm

Mice were initially food deprived to 90% of their body weight and trained in a Pavlovian behavioral paradigm for a total of 7 days with a modular test chamber (17.8 × 15.2 × 18.4 cm; Med Associates), as previously described^76^. Mice were tethered to a photometry cable and habituated to an operant chamber in which there is a house light and pellet receptacle. The house light illuminates as the conditioned stimulus (CS) and sucrose pellets (20 mg, BioServe) are the unconditioned stimulus (US). Each trial consists of 5 s of CS presentation and a single sucrose pellet dropped 7 s after CS onset. The intertrial interval was randomized between 60 and 120 s. The total session was 1 h.

#### Stimulation-evoked release

Mice were restrained at the head in a custom-made head-fixation device^77^ and tethered to a photometry cable. For initial parameter determination experiments, mice received 20-Hz, 5-ms pulse-width laser stimulation in a randomized order varying the stimulus intensity, duration or pulse number, separated by an intertrial interval of 5 min resulting in five trials per mouse per condition, every session. For drug pretreatment experiments, mice were injected with aticaprant at 3 mg per kg body weight or U50,488 at 10 mg per kg body weight body weight i.p using the aforementioned vehicle 30 min before stimulation sessions. Mice received ten trials per mouse with an intertrial interval of 5 min.

#### Fear conditioning paradigm

Mice were placed into a fear conditioning chamber (Med Associates) with a patch cord connected for photometric recordings. A Doric fiber photometry system was used in this study with 465 nm and 405 nm of light (LED, ~30 µW) used for generating the signal and as an isosbestic control, respectively. Each animal received 15 presentations of a 27-s tone (3,000 Hz) co-terminating with a foot shock (0.5 mA for 1.5 s) delivered at 2-min intervals. Each animal received 15 tone–foot shock pairings over the course of 40 min, and the responses for these trials were averaged to create a single trace per animal. Data analysis was performed with custom-written script in MATLAB. In brief, 405-nm traces were fit with a bi-exponential curve, and then the fit was subtracted from the signal to correct for baseline drift. ∆*F/F*% was calculated as [100 × (465 signal − fitted signal)/fitted signal)]. Traces were then *z*-scored. A heat map was plotted using a custom MATLAB script by plotting normalized single trials of traces from all animals tested per brain region.

#### Photometry analysis

Custom MATLAB scripts were developed for analyzing fiber photometry data in the context of mouse behavior. The isosbestic 405-nm excitation control signal was subtracted from the 470-nm excitation signal to remove movement artifacts. Baseline drift was evident in the signal due to slow photobleaching artifacts, particularly during the first several minutes of each recording session. A double exponential curve was fit to the raw trace and subtracted to correct for baseline drift. After baseline correction, the photometry trace was *z*-scored relative to the mean and standard deviation of the session. The post-processed fiber photometry signal was analyzed in the context of animal behavior during Pavlovian conditioning and operant task performance. Pearson correlations, one-sample *t*-tests, two-sample *t*-tests and two-way ANOVAs were performed using standard MATLAB functions ‘corr’, ‘ttest’, ‘ttest2’ and ‘anovan’, respectively. Peak, mean and minimum fluorescence was determined during predetermined time windows for the injection period (0–5 min), reward period (5–20 s), release period (0–20 s) or artifact period (0–20 s) subtracted from peak, mean or minimum fluorescence values in a baseline window (−5 to 0 s). Code that supports the analysis will be made available from the corresponding author upon reasonable request.

#### Perfusion and histology

The stock Avertin (tribromoethanol) was made by mixing 10 g of 2,2,2-tribromoethyl alcohol and 10 ml of tert-amyl alcohol. The working stock was diluted to 1.2% (vol/vol) with water and shielded from light. Animals were euthanized with 125 mg per kg body weight 1.2% Avertin (i.p.) followed by transcardial perfusion with ice-cold 1× PBS and subsequently perfused with ice-cold 4% paraformaldehyde in 1× PBS. After extraction of the mouse brains, samples were post-fixed in 4% paraformaldehyde at 4 °C overnight. The mouse brains were cryo-protected by immersion in 10% sucrose in a 1× PBS solution overnight. Samples were next placed in 30% sucrose in a 1× PBS solution for >1 day, before embedding the samples in O.C.T. Samples were then transferred to a −80 °C freezer for long-term storage or were sliced into 50-µm sections on a cryostat (Leica Biosystems) for histology. Histology samples were imaged on a Leica Stellaris 8 confocal microscope.

### Quantification and statistical analysis

Treatments were randomized, and the data were analyzed by experimenters blinded to the treatment conditions. No statistical methods were used to predetermine sample sizes, but our sample sizes are similar to those reported in previous publications^35,54,56,57,59^. Data distribution was assumed to be normal, but this was not formally tested. Statistical analyses were performed using GraphPad Prism 9 unless noted otherwise. Measurements are taken from distinct samples, and the sample size is indicated as *n* numbers. All comparisons were planned before performing each experiment. A *P* < 0.05 was considered significant. Data are represented as mean ± s.e.m., unless otherwise noted, with asterisks indicating significance levels (*P < 0.05, **P < 0.01, ***P < 0.001 and ****P < 0.0001). No animals or data points were excluded from the analysis.

### Materials availability

The following plasmids have been deposited in Addgene: pCMV- κLight1.3 (201223), pCMV- δLight (201224) and pCMV- µLight (201225). The following viral constructs have been deposited at UNC Neurotools: pAAV-*hSyn*-κLight1.3 (NT-23-888), pAAV-CAG-DIO-κLight1.3a (NT-23-724) and pAAV-*hSyn*-δLight (NT-23-485). κLight1.3, δLight and µLight stable cell lines will be available upon request via MTA with UCD.

### Inclusion and ethics statement

In adherence to the principles outlined in the Global Code of Conduct for Research in Resource-Poor Settings, this study engaged local researchers in all phases, ensuring local relevance and shared ownership of data and intellectual property. Local ethical approval was obtained, and roles were clearly defined and agreed upon with local partners to foster capacity building. This research was conducted with high ethical standards, prioritizing the safety and well-being of all participants, and incorporating benefit-sharing measures for the use of local resources and knowledge.

### Reporting summary

Further information on research design is available in the Nature Portfolio Reporting Summary linked to this article.

## Online content

Any methods, additional references, Nature Portfolio reporting summaries, source data, extended data, supplementary information, acknowledgements, peer review information; details of author contributions and competing interests; and statements of data and code availability are available at 10.1038/s41593-024-01697-1.

## Supplementary information

Reporting Summary

Supplementary Table 1 Supplementary Table 1: Key reagent and resources table.

## Figures and Tables

**Extended Data Table 1:**
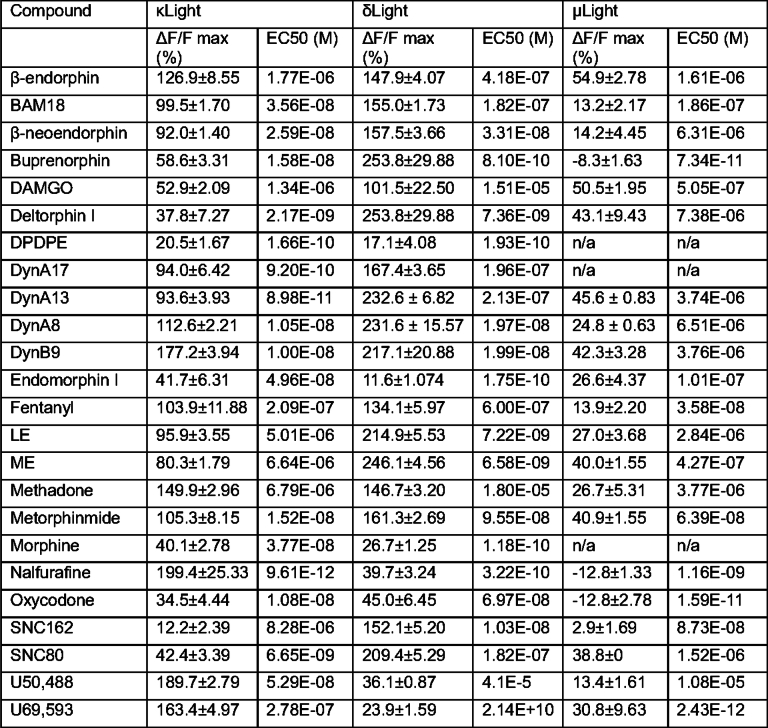
Drug activation and affinity parameters in κLight, δLight, and μLight cells

Dose-response assays from (1pM–100 μM) of each compound screened on κLight, δLight, μLight stable cell lines. ΔF/F max values are sensor responses at 100 μM of each compound. EC50 values were calculated with the mean of ΔF/F values at each concentration of compounds. Data are represented by mean ± SEM, n/a = not applicable.

**Extended Data Table 2:**
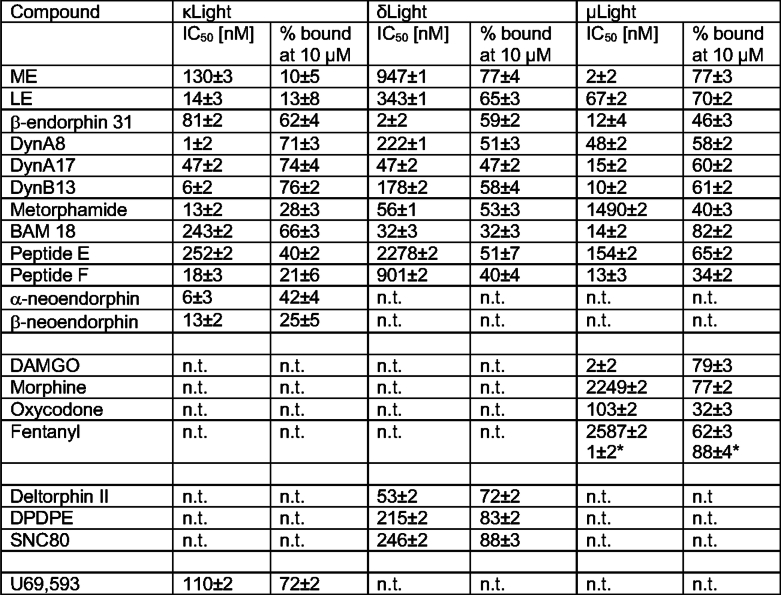
Displacement binding parameters in κLight, δLight, and μLight cells

Displacement binding assays were carried out with membranes (100 μg) from μLight, δLight or κLight cells, [3H] diprenorphine (3 nM final concentration) and different ligands (0- 10 μM final concentration) as described in Methods. Data are Mean ± SE of 3 experiments in triplicate. Binding in the absence of cold ligand was taken as 100% bound. Binding in the presence of 10 μM ligand was taken as non-specific binding. % Specific binding at 10 μM = Total binding – non-specific binding. n.t.= not tested. * Displacement binding assays were carried out with CHO-MOR membranes (100 μg).

## Data Availability

The following public dataset was used to support this study: Allen Brain Atlas ISH data (https://mouse.brain-map.org/). All source data present in this manuscript are available from https://github.com/lintianlab/OpioidSensors/tree/main/0-SourceData/.

## Code availability

All custom MATLAB codes are available from https://github.com/lintianlab/OpioidSensors/tree/main/1-Codes/.

## References

[1] Greco, M. A. et al. Opioidergic projections to sleep-active neurons in the ventrolateral preoptic nucleus. *Brain Res.* **1245**, 96–107 (2008). 18840417 10.1016/j.brainres.2008.09.043 PMC2753822

[2] Hökfelt, T. et al. Neuropeptide and small transmitter coexistence: fundamental studies and relevance to mental illness. *Front. Neural Circuits* **12**, 106 (2018). 30627087 10.3389/fncir.2018.00106 PMC6309708

[3] Holden, J. E., Jeong, Y. & Forrest, J. M. The endogenous opioid system and clinical pain management. *AACN Clin. Issues* **16**, 291–301 (2005). 16082232 10.1097/00044067-200507000-00003

[4] Klenowski, P., Morgan, M. & Bartlett, S. E. The role of δ-opioid receptors in learning and memory underlying the development of addiction. *Br. J. Pharmacol.* **172**, 297–310 (2015). 24641428 10.1111/bph.12618 PMC4292947

[5] Nummenmaa, L. & Tuominen, L. Opioid system and human emotions. *Br. J. Pharmacol.* **175**, 2737–2749 (2018). 28394427 10.1111/bph.13812 PMC6016642

[6] Russo, A. F. Overview of neuropeptides: awakening the senses? *Headache* **57**, 37–46 (2017). 28485842 10.1111/head.13084 PMC5424629

[7] Fricker, L. D. Analysis of mouse brain peptides using mass spectrometry-based peptidomics: implications for novel functions ranging from non-classical neuropeptides to microproteins. *Mol. Biosyst.* **6**, 1355–1365 (2010). 20428524 10.1039/c003317k PMC2902593

[8] van den Pol, A. N. Neuropeptide transmission in brain circuits. *Neuron* **76**, 98–115 (2012). 23040809 10.1016/j.neuron.2012.09.014 PMC3918222

[9] Burns, J. A. et al. Molecular imaging of opioid and dopamine systems: insights into the pharmacogenetics of opioid use disorders. *Front. Psychiatry* **10**, 626 (2019). 31620026 10.3389/fpsyt.2019.00626 PMC6759955

[10] Corder, G., Castro, D. C., Bruchas, M. R. & Scherrer, G. Endogenous and exogenous opioids in pain. *Annu. Rev. Neurosci.* **41**, 453–473 (2018). 29852083 10.1146/annurev-neuro-080317-061522 PMC6428583

[11] Franchi, S., Moschetti, G., Amodeo, G. & Sacerdote, P. Do all opioid drugs share the same immunomodulatory properties? A review from animal and human studies. *Front. Immunol.* **10**, 2914 (2019). 31921173 10.3389/fimmu.2019.02914 PMC6920107

[12] Higginbotham, J. A., Markovic, T., Massaly, N. & Morón, J. A. Endogenous opioid systems alterations in pain and opioid use disorder. *Front. Syst. Neurosci.* **16**, 1014768 (2022). 36341476 10.3389/fnsys.2022.1014768 PMC9628214

[13] Le Merrer, J., Becker, J. A., Befort, K. & Kieffer, B. L. Reward processing by the opioid system in the brain. *Physiol. Rev.* **89**, 1379–1412 (2009). 19789384 10.1152/physrev.00005.2009 PMC4482114

[14] Machelska, H. & Celik, M. Ö. Advances in achieving opioid analgesia without side effects. *Front. Pharmacol.* **9**, 1388 (2018). 30555325 10.3389/fphar.2018.01388 PMC6282113

[15] Pathan, H. & Williams, J. Basic opioid pharmacology: an update. *Br. J. Pain* **6**, 11–16 (2012). 26516461 10.1177/2049463712438493 PMC4590096

[16] Gomes, I. et al. Biased signaling by endogenous opioid peptides. *Proc. Natl Acad. Sci. USA* **117**, 11820–11828 (2020). 32393639 10.1073/pnas.2000712117 PMC7261131

[17] Machelska, H. & Celik, M. Ö. Opioid receptors in immune and glial cells—implications for pain control. *Front. Immunol.* **11**, 300 (2020). 32194554 10.3389/fimmu.2020.00300 PMC7064637

[18] Mansour, A. et al. Mu, delta, and kappa opioid receptor mRNA expression in the rat CNS: an in situ hybridization study. *J. Comp. Neurol.* **350**, 412–438 (1994). 7884049 10.1002/cne.903500307

[19] McGinty, J. F., van der Kooy, D. & Bloom, F. E. The distribution and morphology of opioid peptide immunoreactive neurons in the cerebral cortex of rats. *J. Neurosci.* **4**, 1104–1117 (1984). 6143786 10.1523/JNEUROSCI.04-04-01104.1984 PMC6564782

[20] van Steenbergen, H., Eikemo, M. & Leknes, S. The role of the opioid system in decision making and cognitive control: a review. *Cogn. Affect. Behav. Neurosci.* **19**, 435–458 (2019). 30963411 10.3758/s13415-019-00710-6 PMC6599188

[21] Kosten, T. R. & George, T. P. The neurobiology of opioid dependence: implications for treatment. *Sci. Pract. Perspect.* **1**, 13–20 (2002). 18567959 10.1151/spp021113 PMC2851054

[22] Al-Hasani, R. & Bruchas, M. R. Molecular mechanisms of opioid receptor-dependent signaling and behavior. *Anesthesiology* **115**, 1363–1381 (2011). 22020140 10.1097/ALN.0b013e318238bba6 PMC3698859

[23] Bruchas, M. R. et al. Stress-induced p38 mitogen-activated protein kinase activation mediates kappa-opioid-dependent dysphoria. *J. Neurosci.* **27**, 11614–11623 (2007). 17959804 10.1523/JNEUROSCI.3769-07.2007 PMC2481272

[24] Hoyer, D. & Bartfai, T. in *Encyclopedia of Neuroscience* (ed. Squire, L. R.) 801–810 (Academic Press, 2009).

[25] Fricker, L. D., Margolis, E. B., Gomes, I. & Devi, L. A. Five decades of research on opioid peptides: current knowledge and unanswered questions. *Mol. Pharmacol.* **98**, 96–108 (2020). 32487735 10.1124/mol.120.119388 PMC7330675

[26] Castro, D. C. et al. An endogenous opioid circuit determines state-dependent reward consumption. *Nature* **598**, 646–651 (2021). 34646022 10.1038/s41586-021-04013-0 PMC8858443

[27] Ma, X. et al. In vivo photopharmacology with a caged mu opioid receptor agonist drives rapid changes in behavior. *Nat. Methods* **20**, 682–685 (2023). 36973548 10.1038/s41592-023-01819-w PMC10569260

[28] Smith, S. J. et al. Single-cell transcriptomic evidence for dense intracortical neuropeptide networks. *eLife* **8**, e47889 (2019). 31710287 10.7554/eLife.47889 PMC6881117

[29] Banghart, M. R. & Sabatini, B. L. Photoactivatable neuropeptides for spatiotemporally precise delivery of opioids in neural tissue. *Neuron* **73**, 249–259 (2012). 22284180 10.1016/j.neuron.2011.11.016 PMC3282187

[30] Al-Hasani, R. et al. In vivo detection of optically-evoked opioid peptide release. *eLife* **7**, e36520 (2018). 30175957 10.7554/eLife.36520 PMC6135606

[31] Mitsui, S., Saito, M., Hayashi, K., Mori, K. & Yoshihara, Y. A novel phenylalanine-based targeting signal directs telencephalin to neuronal dendrites. *J. Neurosci.* **25**, 1122–1131 (2005). 15689548 10.1523/JNEUROSCI.3853-04.2005 PMC6725959

[32] Stockklausner, C., Ludwig, J., Ruppersberg, J. P. & Klöcker, N. A sequence motif responsible for ER export and surface expression of Kir2.0 inward rectifier K^+^ channels. *FEBS Lett.* **493**, 129–133 (2001). 11287009 10.1016/S0014-5793(01)02286-4

[33] Lim, S. T., Antonucci, D. E., Scannevin, R. H. & Trimmer, J. S. A novel targeting signal for proximal clustering of the Kv2.1 K^+^ channel in hippocampal neurons. *Neuron* **25**, 385–397 (2000). 10719893 10.1016/S0896-6273(00)80902-2

[34] Tandon, N., Thakkar, K. N., LaGory, E. L., Liu, Y. & Giaccia, A. J. Generation of stable expression mammalian cell lines using lentivirus. *Bio Protoc.* **8**, e3073 (2018). 30505888 10.21769/BioProtoc.3073 PMC6261361

[35] Dong, C. et al. Psychedelic-inspired drug discovery using an engineered biosensor. *Cell* **184**, 2779–2792.e18 (2021). 33915107 10.1016/j.cell.2021.03.043 PMC8122087

[36] Nichols, A. L. et al. Fluorescence activation mechanism and imaging of drug permeation with new sensors for smoking-cessation ligands. *eLife* **11**, e74648 (2022). 34982029 10.7554/eLife.74648 PMC8820738

[37] Gomes, I., Filipovska, J. & Devi, L. A. Opioid receptor oligomerization. Detection and functional characterization of interacting receptors. *Methods Mol. Med.* **84**, 157–183 (2003). 12703323 10.1385/1-59259-379-8:157

[38] Gomes, I., Ijzerman, A. P., Ye, K., Maillet, E. L. & Devi, L. A. G-protein-coupled receptor heteromerization: a role in allosteric modulation of ligand binding. *Mol. Pharmacol.* **79**, 1044–1052 (2011). 21415307 10.1124/mol.110.070847 PMC3102551

[39] Banghart, M. R., He, X. J. & Sabatini, B. L. A caged enkephalin optimized for simultaneously probing mu and delta opioid receptors. *ACS Chem. Neurosci.* **9**, 684–690 (2018). 29266926 10.1021/acschemneuro.7b00485 PMC5906201

[40] He, X. J. et al. Convergent, functionally independent signaling by mu and delta opioid receptors in hippocampal parvalbumin interneurons. *eLife* **10**, e69746 (2021). 34787079 10.7554/eLife.69746 PMC8716102

[41] Toll, L. et al. Standard binding and functional assays related to medications development division testing for potential cocaine and opiate narcotic treatment medications. *NIDA Res. Monogr.* **178**, 440–466 (1998). 9686407

[42] Ino, D., Tanaka, Y., Hibino, H. & Nishiyama, M. A fluorescent sensor for real-time measurement of extracellular oxytocin dynamics in the brain. *Nat. Methods* **19**, 1286–1294 (2022). 36138174 10.1038/s41592-022-01597-x PMC9550624

[43] Shirayama, Y. et al. Stress increases dynorphin immunoreactivity in limbic brain regions and dynorphin antagonism produces antidepressant-like effects. *J. Neurochem.* **90**, 1258–1268 (2004). 15312181 10.1111/j.1471-4159.2004.02589.x

[44] Wagner, J. J., Terman, G. W. & Chavkin, C. Endogenous dynorphins inhibit excitatory neurotransmission and block LTP induction in the hippocampus. *Nature* **363**, 451–454 (1993). 8099201 10.1038/363451a0 PMC2096733

[45] Weisskopf, M. G., Zalutsky, R. A. & Nicoll, R. A. The opioid peptide dynorphin mediates heterosynaptic depression of hippocampal mossy fibre synapses and modulates long-term potentiation. *Nature* **362**, 423–427 (1993). 8096624 10.1038/362423a0

[46] Romero-Picó, A. et al. Hypothalamic κ-opioid receptor modulates the orexigenic effect of ghrelin. *Neuropsychopharmacology* **38**, 1296–1307 (2013). 23348063 10.1038/npp.2013.28 PMC3656373

[47] Al-Hasani, R. et al. Distinct subpopulations of nucleus accumbens dynorphin neurons drive aversion and reward. *Neuron* **87**, 1063–1077 (2015). 26335648 10.1016/j.neuron.2015.08.019 PMC4625385

[48] Castro, D. C. & Berridge, K. C. Opioid hedonic hotspot in nucleus accumbens shell: mu, delta, and kappa maps for enhancement of sweetness ‘liking’ and ‘wanting’. *J. Neurosci.* **34**, 4239–4250 (2014). 24647944 10.1523/JNEUROSCI.4458-13.2014 PMC3960467

[49] Stuber, G. D. et al. Excitatory transmission from the amygdala to nucleus accumbens facilitates reward seeking. *Nature* **475**, 377–380 (2011). 21716290 10.1038/nature10194 PMC3775282

[50] Tejeda, H. A. et al. Pathway- and cell-specific kappa-opioid receptor modulation of excitation-inhibition balance differentially gates D1 and D2 accumbens neuron activity. *Neuron* **93**, 147–163 (2017). 28056342 10.1016/j.neuron.2016.12.005 PMC5808882

[51] Page, S. et al. Behavioral pharmacology of novel kappa opioid receptor antagonists in rats. *Int. J. Neuropsychopharmacol.* **22**, 735–745 (2019). 31613314 10.1093/ijnp/pyz054 PMC7145521

[52] Dong, C. et al. Fluorescence imaging of neural activity, neurochemical dynamics, and drug-specific receptor conformation with genetically encoded sensors. *Annu. Rev. Neurosci.* **45**, 273–294 (2022). 35316611 10.1146/annurev-neuro-110520-031137 PMC9940643

[53] Wu, Z., Lin, D. & Li, Y. Pushing the frontiers: tools for monitoring neurotransmitters and neuromodulators. *Nat. Rev. Neurosci.* **23**, 257–274 (2022). 35361961 10.1038/s41583-022-00577-6 PMC11163306

[54] Borden, P. M. et al. A fast genetically encoded fluorescent sensor for faithful in vivo acetylcholine detection in mice, fish, worms and flies. Preprint at *bioRxiv*10.1101/2020.02.07.939504 (2020).

[55] Jing, M. et al. An optimized acetylcholine sensor for monitoring in vivo cholinergic activity. *Nat. Methods* **17**, 1139–1146 (2020). 32989318 10.1038/s41592-020-0953-2 PMC7606762

[56] Patriarchi, T. et al. Ultrafast neuronal imaging of dopamine dynamics with designed genetically encoded sensors. *Science* **360**, eaat4422 (2018). 29853555 10.1126/science.aat4422 PMC6287765

[57] Patriarchi, T. et al. An expanded palette of dopamine sensors for multiplex imaging in vivo. *Nat. Methods* **17**, 1147–1155 (2020). 32895537 10.1038/s41592-020-0936-3 PMC8169200

[58] Sun, F. et al. Next-generation GRAB sensors for monitoring dopaminergic activity in vivo. *Nat. Methods* **17**, 1156–1166 (2020). 33087905 10.1038/s41592-020-00981-9 PMC7648260

[59] Unger, E. K. et al. Directed evolution of a selective and sensitive serotonin sensor via machine learning. *Cell* **183**, 1986–2002 (2020). 33333022 10.1016/j.cell.2020.11.040 PMC8025677

[60] Wan, J. et al. A genetically encoded sensor for measuring serotonin dynamics. *Nat. Neurosci.* **24**, 746–752 (2021). 33821000 10.1038/s41593-021-00823-7 PMC8544647

[61] Duffet, L. et al. A genetically encoded sensor for in vivo imaging of orexin neuropeptides. *Nat. Methods* **19**, 231–241 (2022). 35145320 10.1038/s41592-021-01390-2 PMC8831244

[62] Qian, T. et al. A genetically encoded sensor measures temporal oxytocin release from different neuronal compartments. *Nat. Biotechnol.* **41**, 944–957 (2023). 36593404 10.1038/s41587-022-01561-2 PMC11182738

[63] Wang, H. et al. A tool kit of highly selective and sensitive genetically encoded neuropeptide sensors. *Science*10.1126/science.abq8173 (2023). 10.1126/science.abq8173 PMC11205257 37972184

[64] Sounier, R. et al. Propagation of conformational changes during μ-opioid receptor activation. *Nature* **524**, 375–378 (2015). 26245377 10.1038/nature14680 PMC4820006

[65] Drake, C. T. et al. Dynorphin opioids present in dentate granule cells may function as retrograde inhibitory neurotransmitters. *J. Neurosci.* **14**, 3736–3750 (1994). 7911518 10.1523/JNEUROSCI.14-06-03736.1994 PMC6576943

[66] Nicholson, C. & Tao, L. Hindered diffusion of high molecular weight compounds in brain extracellular microenvironment measured with integrative optical imaging. *Biophys. J.* **65**, 2277–2290 (1993). 7508761 10.1016/S0006-3495(93)81324-9 PMC1225970

[67] Xiong, H. et al. Probing neuropeptide volume transmission in vivo by simultaneous near-infrared light-triggered release and optical sensing. *Angew. Chem. Int. Ed.*10.1002/anie.202206122 (2022). 10.1002/anie.202206122 PMC9388559 35723610

[68] Atwood, B. K., Kupferschmidt, D. A. & Lovinger, D. M. Opioids induce dissociable forms of long-term depression of excitatory inputs to the dorsal striatum. *Nat. Neurosci.* **17**, 540–548 (2014). 24561996 10.1038/nn.3652 PMC4163916

[69] Quan, J. & Tian, J. Circular polymerase extension cloning for high-throughput cloning of complex and combinatorial DNA libraries. *Nat. Protoc.* **6**, 242–251 (2011). 21293463 10.1038/nprot.2010.181

[70] Irannejad, R. et al. Conformational biosensors reveal GPCR signalling from endosomes. *Nature* **495**, 534–538 (2013). 23515162 10.1038/nature12000 PMC3835555

[71] Chen, T. W. et al. Ultrasensitive fluorescent proteins for imaging neuronal activity. *Nature* **499**, 295–300 (2013). 23868258 10.1038/nature12354 PMC3777791

[72] Tian, L. et al. Imaging neural activity in worms, flies and mice with improved GCaMP calcium indicators. *Nat. Methods* **6**, 875–881 (2009). 19898485 10.1038/nmeth.1398 PMC2858873

[73] Janetzko, J. et al. Membrane phosphoinositides regulate GPCR–β-arrestin complex assembly and dynamics. *Cell* **185**, 4560–4573.e19 (2022). 36368322 10.1016/j.cell.2022.10.018 PMC10030194

[74] Thorne, R. G. & Nicholson, C. In vivo diffusion analysis with quantum dots and dextrans predicts the width of brain extracellular space. *Proc. Natl Acad. Sci. USA* **103**, 5567–5572 (2006). 16567637 10.1073/pnas.0509425103 PMC1459394

[75] Ting, J. T., Daigle, T. L., Chen, Q. & Feng, G. in *Patch-Clamp Methods and Protocols* (eds Martina, M. & Taverna, S.) 221–242 (Springer, 2014).

[76] Al-Hasani, R. et al. Author correction: ventral tegmental area GABAergic inhibition of cholinergic interneurons in the ventral nucleus accumbens shell promotes reward reinforcement. *Nat. Neurosci.* **24**, 1501 (2021). 34504334 10.1038/s41593-021-00928-z

[77] Piantadosi, S. C. et al. Hyperactivity of indirect pathway-projecting spiny projection neurons promotes compulsive behavior. *Nat. Commun.*10.1038/s41467-024-48331-z (2024). 10.1038/s41467-024-48331-z PMC11126597 38789416

