# Supplementary material for: Unlocking opioid neuropeptide dynamics with genetically encoded biosensors
Source: Nat Neurosci. 2024 Jul 15;27(9):1844–57. doi: 10.1038/s41593-024-01697-1 (PMC11374718; doi:10.1038/s41593-024-01697-1)
Supplement: Supplementary file 1 — Reporting Summary [file 41593_2024_1697_MOESM1_ESM.pdf]

nature portfolio

Corresponding author(s): Lin Tian, Matthew R. Banghart, Michael R. Bruchas

Last updated by author(s): May 14, 2024

Reporting Summary

Nature Portfolio wishes to improve the reproducibility of the work that we publish. This form provides structure for consistency and transparency in reporting. For further information on Nature Portfolio policies, see our Editorial Policies and the Editorial Policy Checklist.

Statistics

For all statistical analyses, confirm that the following items are present in the figure legend, table legend, main text, or Methods section.

n/a	Confirmed	
☐	☒	The exact sample size (*n*) for each experimental group/condition, given as a discrete number and unit of measurement
☐	☒	A statement on whether measurements were taken from distinct samples or whether the same sample was measured repeatedly
☐	☒	The statistical test(s) used AND whether they are one- or two-sided<br>*Only common tests should be described solely by name; describe more complex techniques in the Methods section.*
☒	☐	A description of all covariates tested
☐	☒	A description of any assumptions or corrections, such as tests of normality and adjustment for multiple comparisons
☐	☒	A full description of the statistical parameters including central tendency (e.g. means) or other basic estimates (e.g. regression coefficient) AND variation (e.g. standard deviation) or associated estimates of uncertainty (e.g. confidence intervals)
☐	☒	For null hypothesis testing, the test statistic (e.g. *F*, *t*, *r*) with confidence intervals, effect sizes, degrees of freedom and *P* value noted<br>*Give P values as exact values whenever suitable.*
☒	☐	For Bayesian analysis, information on the choice of priors and Markov chain Monte Carlo settings
☒	☐	For hierarchical and complex designs, identification of the appropriate level for tests and full reporting of outcomes
☒	☐	Estimates of effect sizes (e.g. Cohen's *d*, Pearson's *r*), indicating how they were calculated

Our web collection on statistics for biologists contains articles on many of the points above.

Software and code

Policy information about availability of computer code

Data collection	We have provided a code availability statement. Fiber photometry data collected by Doric Neuroscience Studio V5.4.1.12, Fear behaviors are controlled by Video Freeze V3.0.0.0, Microconfocal HEK cell imaging data was collected by MetaXpress V6.6.3.55, two-photon data was collected by ScanImage 5 run by Matlab R2013b.
Data analysis	GraphPad Prism 9, Matlab R2022a, and Jupyter Notebook 6.4.6, ImageJ Version:2.1.0/1.53c. Microsoft Excel 16.72, Illustrator 2024. All custom MATLAB codes are available from https://github.com/lintianlab/OpioidSensors/tree/main/1-Codes.

For manuscripts utilizing custom algorithms or software that are central to the research but not yet described in published literature, software must be made available to editors and reviewers. We strongly encourage code deposition in a community repository (e.g. GitHub). See the Nature Portfolio guidelines for submitting code & software for further information.

Data

Policy information about availability of data

All manuscripts must include a data availability statement. This statement should provide the following information, where applicable:

- Accession codes, unique identifiers, or web links for publicly available datasets
- A description of any restrictions on data availability
- For clinical datasets or third party data, please ensure that the statement adheres to our policy

The following public dataset were used to support this study: Allen Brain Atlas ISH data (https://mouse.brain-map.org). All source data present in this manuscript are available from https://github.com/lintianlab/OpioidSensors/tree/main/0-SourceData.

Research involving human participants, their data, or biological material

Policy information about studies with human participants or human data. See also policy information about sex, gender (identity/presentation), and sexual orientation and race, ethnicity and racism.

Reporting on sex and gender	n/a
Reporting on race, ethnicity, or other socially relevant groupings	n/a
Population characteristics	n/a
Recruitment	n/a
Ethics oversight	n/a

Note that full information on the approval of the study protocol must also be provided in the manuscript.

Field-specific reporting

Please select the one below that is the best fit for your research. If you are not sure, read the appropriate sections before making your selection.

☒ Life sciences ☐ Behavioural & social sciences ☐ Ecological, evolutionary & environmental sciences

For a reference copy of the document with all sections, see nature.com/documents/nr-reporting-summary-flat.pdf

Life sciences study design

All studies must disclose on these points even when the disclosure is negative.

Sample size	No statistical methods were used to predetermine sample size. However, sample sizes are consistent with those reported in previous publications (35,54-60).
Data exclusions	No data were excluded from this study
Replication	Each and every experiment was performed with at least n = 3 biological independent cultures or animal subjects. Sample size for each experiment is indicated in the figure legends. All attempts were successful.
Randomization	Animals and cultures were randomly assigned for transduction and transfection with the different constructs described in the manuscript. Drug testing on stable cell lines are randomly chosen wells on imaging plates to ensure each biological replicates have no plate location influence on results.
Blinding	Drug screening experiments are done with two person. One prepare the compound treatment plates, and the other do the imaging blindly. Data analysis were done with matlab script to analyze uniformly for data generation. In vivo experiments are done blindly without knowing which animal is expressing which construct until the data is analyzed.

Reporting for specific materials, systems and methods

We require information from authors about some types of materials, experimental systems and methods used in many studies. Here, indicate whether each material, system or method listed is relevant to your study. If you are not sure if a list item applies to your research, read the appropriate section before selecting a response.

Materials & experimental systems

n/a	Involved in the study
☒	☐ Antibodies
☐	☒ Eukaryotic cell lines
☒	☐ Palaeontology and archaeology
☐	☒ Animals and other organisms
☒	☐ Clinical data
☒	☐ Dual use research of concern
☒	☐ Plants

Methods

n/a	Involved in the study
☒	☐ ChIP-seq
☒	☐ Flow cytometry
☒	☐ MRI-based neuroimaging

Eukaryotic cell lines

Policy information about cell lines and Sex and Gender in Research

Cell line source(s)	HEK293T (ATCC, CRL-3126, https://www.atcc.org/products/crl-3216). Three sensor expressing stable cell lines are generated based on HEK293T cells from this study.
Authentication	These cell lines were not authenticated
Mycoplasma contamination	These cell lines were not tested for mycoplasma contamination.
Commonly misidentified lines (See ICLAC register)	No commonly misidentified cell lines were used in the study

Animals and other research organisms

Policy information about studies involving animals; ARRIVE guidelines recommended for reporting animal research, and Sex and Gender in Research

Laboratory animals	C57/B6J mice (postnatal day 0-3, 8-10 weeks, or 12-16 weeks) were used in this study as described in the manuscript. Adult (25-35 g), 12-16 week old KOR-Cre mice were used for optostimulation experiments. Animals are housed in ventilated home cages with at most five mice per cage. Animals are housed in vivarium with 12 hour light and 12 hour dark standard light cycle, with temperature of 68 – 79 °F and humidity 30 - 70%.
Wild animals	We did not use wild animals.
Reporting on sex	Both male and female mice were used in this study.
Field-collected samples	We did not use field-collected samples.
Ethics oversight	All housing and experimental procedures involving animals were approved by the Institutional Animal Care and Use Committee (IACUC) at the University of California, Davis, the University of California San Diego, the University of Washington, the University of Iowa Cold Spring Harbor Laboratory, the National Institute of Mental Health, or Icahn School of Medicine, and adhered to principles described in the National Institutes of Health Guide for the Care and Use of Laboratory Animals. The University of California, Davis, the University of California San Diego, the University of Washington, the University of Iowa, Cold Spring Harbor Laboratory, the National Institute of Mental Health, and Icahn School of Medicine are accredited by the Association for Assessment and Accreditation of Laboratory Animal Care International (AAALAC).

Note that full information on the approval of the study protocol must also be provided in the manuscript.
